# Enhanced corrosion resistance by engineering crystallography on metals

**DOI:** 10.1038/s41467-022-28368-8

**Published:** 2022-02-07

**Authors:** X. X. Wei, B. Zhang, B. Wu, Y. J. Wang, X. H. Tian, L. X. Yang, E. E. Oguzie, X. L. Ma

**Affiliations:** 1 Shenyang National Laboratory for Materials Science, Institute of Metal Research, Chinese Academy of Sciences, Wenhua Road 72, Shenyang, 110016 China; 2 School of Materials Science and Engineering, University of Science and Technology of China, Shenyang, 110016 China; 3 Bay Area Center for Electron Microscopy, Songshan Lake Materials Laboratory, Dongguan, Guangdong 523808 China; 4 Africa Centre of Excellence in Future Energies and Electrochemical Systems, Federal University of Technology Owerri, P.M.B 1526 Owerri, Imo State Nigeria; 5 State Key Lab of Advanced Processing and Recycling on Non-ferrous Metals, Lanzhou University of Technology, Lanzhou, 730050 China

**Keywords:** Metals and alloys, Characterization and analytical techniques

## Abstract

Nanometer-thick passive films, which impart superior corrosion resistance to metals, are degraded in long-term service; they are also susceptible to chloride-induced localized attack. Here we show, by engineering crystallographic configurations upon metal matrices adjacent to their passive films, we obtain great enhancement of corrosion resistance of FeCr15Ni15 single crystal in sulphuric acid, with activation time up to two orders of magnitude longer than that of the non-engineered counterparts. Meanwhile, engineering crystallography decreases the passive current density and shifts the pitting potential to noble values. Applying anodic polarizations under a transpassivation potential, we make the metal matrices underneath the transpassive films highly uneven with {111}-terminated configurations, which is responsible for the enhancement of corrosion resistance. The transpassivation strategy also works in the commercial stainless steels where both grain interior and grain boundaries are rebuilt into the low-energy configurations. Our results demonstrate a technological implication in the pretreatment process of anti-corrosion engineering.

## Abstract (web summary)

Passive films on metal surfaces provide better corrosion resistance, but they can degrade in long-term service. Here the authors demonstrate a strategy to engineer crystallographic configuration at the metal/film interface to further improve corrosion resistance.

## Introduction

Corrosion is the scourge in our metal-based civilization. Corrosion-induced material degradation annually accounts for at least 2–3% of gross national product for an industrialized economy^1^. Austenitic stainless steels, a typical class of passive metals, are known to be widely deployed in service in industrial sectors like the chemical, petrochemical, and nuclear industry, in which they are often exposed to acidic and chloride-containing media. For passive metals, which are known to have superior corrosion resistance due to the presence of compact and protective surficial passive films, the nano-scale passive films still undergo degradation in acid media and make the metals susceptible to pitting corrosion in chloride-containing media. It is therefore not surprising that corrosion resistance of materials has in the past several decades, been correlated directly to passive film stability. Accordingly, great attention has been paid to the nature of the passive film itself, wherein the film thickness, Cr/Fe ratio, higher-valence metal oxide, and defect concentration are considered as the key indicators of the stability of passive films^2–8^. In contrast, the possible role of the interface between the passive film and the metal (Me/F), particularly the atomic configurations therein, is rarely taken into account in evaluating passive film stability.

Although the Me/F interface does not directly come in contact with the corrosive media, the interface could be involved in the electrode reactions during metal degradation. As early as the 1930s, it was found that an anodically-formed oxide film on unalloyed iron could be thinned or even completely eliminated in sulfuric acid; whereas, the same film could survive for considerably longer periods without dissolution when it was transferred from the metal to plastic support with no electrical contact with the metal^9,10^. Such rapid dissolution of the passive film in contact with a metal matrix had been rationalized via a reductive dissolution model, wherein the cathodic reaction destroys the film, while the anodic reaction dissolves the metal matrix, as illustrated below^10–12^.

1 $${{{{{{\rm{Fe}}}}}}}_{2}{{{{{{\rm{O}}}}}}}_{3}\,+\,{{{{{{\rm{6H}}}}}}}^{+}\,+\,{{{{{\rm{2e}}}}}}\to {{{{{{\rm{2Fe}}}}}}}^{2+}\,+\,{{{{{{\rm{3H}}}}}}}_{2}{{{{{\rm{O}}}}}}$$

2 $${{{{{\rm{Fe}}}}}}\to {{{{{{\rm{2Fe}}}}}}}^{2+}\,+\,{{{{{\rm{2e}}}}}}$$

Accordingly, avoiding or retarding the reductive dissolution is suggested to be one of the ways to improve the resistance of ferrous material to acid. Alloying chromium has been well known to be an effective way^11^, which can form an oxide film and is less stable than iron in the divalent state. The reduction of Cr_2_O_3_ to Cr^2+^ would need even a lower potential than the reduction of oxygen, and thus chromium oxide is difficult to experience reductive dissolution^11^. However, the passive film on the stainless steel is not pure Cr_2_O_3_ but comprised of mixed iron and chromium oxide. The component of chromium oxide highly retards the reductive dissolution of the passive film and thus the Cr content in the passive film is well known to be correlated with the corrosion resistance. In contrast, the iron oxide component of the film would suffer reductive dissolution, which is considered to be the most important process for the stability of stainless steel^13^. Accordingly, developing novel strategies to mitigate the reductive dissolution of the iron oxide component in the passive film of stainless steel would impart improved corrosion resistance.

According to the reductive dissolution model, the electrode reactions during degradation of the surface oxide film in acid media, occur not only at the film but also at the metal surface. This model, therefore, specifies some role of the atomic configurations at the Me/F interface in controlling the reactivity and hence the stability of the passive film and ultimately the corrosion resistance. Electrochemical reactivity is anisotropic with crystallographic orientations^14–20^ due to the different surface energy levels^16^ or/and coordination numbers of surface atoms^21^ at varied crystallographic planes. It is generally believed that the close-packed plane has the lowest surface energy and the most coordination numbers, and therefore it is relatively inactive in electrochemistry. From this viewpoint, improving the stability of a passive film could be achieved by engineering a characteristic interface, wherein the metal matrix is enclosed by the close-packed planes.

In this work, we apply an extremely positive potential of 1.1 V/SCE within the transpassive region to a passivated FeCr15Ni15 single-crystal alloy and a commercial 304 stainless steel and obtain a fluctuating Me/F interface in which the metal is composed of a large number of close-packed {111} planes. We find that the resistance of the passive film to reductive dissolution as well as to pitting corrosion is much improved, expanding the activation time (τ) of the passive film in sulfuric acid up to two orders of magnitude; we also observe a big transpassivation-induced shift of pitting potentials towards more noble values. We thus propose a high-efficiency and low-cost crystallographic engineering procedure for imparting enhanced corrosion resistance to metals.

## Results

### Structural evolution of the Me/F interface during film degradation

**Fig. 1:**
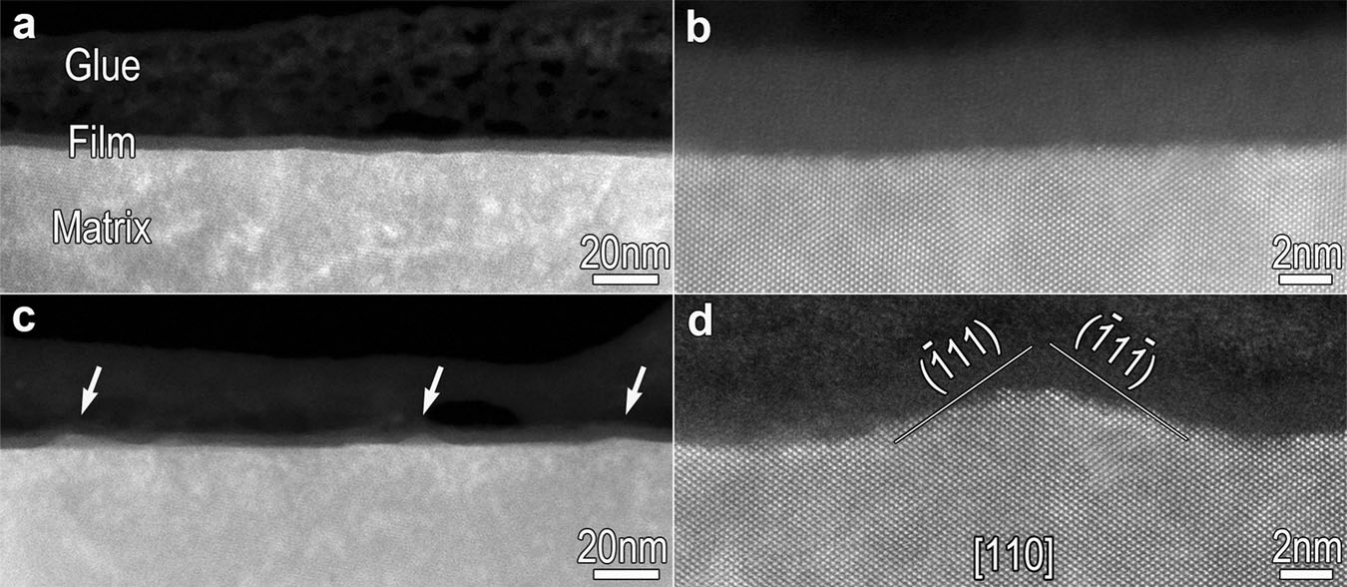
Reductive dissolution of a passive film in acid solution is evidenced experimentally. **a**, **b** HAADF-STEM (**a**) and HRHAADF-STEM (**b**) images showing the sharp, well-defined, and straight Me/F interfaces of the passivated FeCr15Ni15 alloy. **c**, **d** HAADF-STEM (**c**) and HRHAADF-STEM (**d**) images of the passivated specimen on immersion in sulfuric acid (5.6 mol L^−1^) for about 20 min clearly shows that the straight interface in the pristine passive film becomes undulating. The walls of the swell are along the close-packed {111} plane.

Passive films were formed on the (110) plane of FeCr15Ni15 single crystal (Supplementary Fig. 1 and Supplementary Notes 1,  2). In order to clarify the corrosion events occurring during passive film degradation in acid media, we conducted experiments with the passivated single-crystal specimens immersed in sulfuric acid (Supplementary Fig. 2) and closely monitored the structural evolution of the film, paying special attention to the Me/F interface. The high angle annular dark-field scanning transmission electron microscopic (HAADF-STEM) images of the passive films prior to and after the immersion in sulfuric acid, clearly reveal that the sharp, well-defined, and straight interface in the pristine passive film becomes undulating after the immersion in acid solution (Fig. 1 and Supplementary Fig. 3). This observation is symptomatic of matrix dissolution events during the passive film degradation in acid media and provides clear evidence supporting the reductive dissolution model. Meanwhile, we find the walls of the undulating interface to be parallel to the specified {111} crystallographic planes (Fig. 1d and Supplementary Fig. 3e, f), which strongly implies that the dissolution of the matrix is anisotropic with crystallographic orientation, going faster along the [110] than the [111] direction, leaving some intact {111} planes at the Me/F interface. Since the direct dissolution of the metal matrix is considered to be the rate-controlling step in the reductive dissolution model^12,13^, the fast dissolution of the metal atoms at {110} planes then determines the reductive dissolution (degradation) rate of the surficial passive film. Conversely, if we pre-construct the Me/F interface with a large number of {111} planes exposed, the oxidative dissolution of the metal matrix on {111} planes would be effectively retarded and thus the reductive dissolution of the passive film would be significantly suppressed. This means that the structure of the interface plays an important role in passive film degradation, and that film stability can be further improved by atomic-scale engineering to yield an inactive interface.

### Engineering of an interface with a predominance of {111} planes

**Fig. 2:**
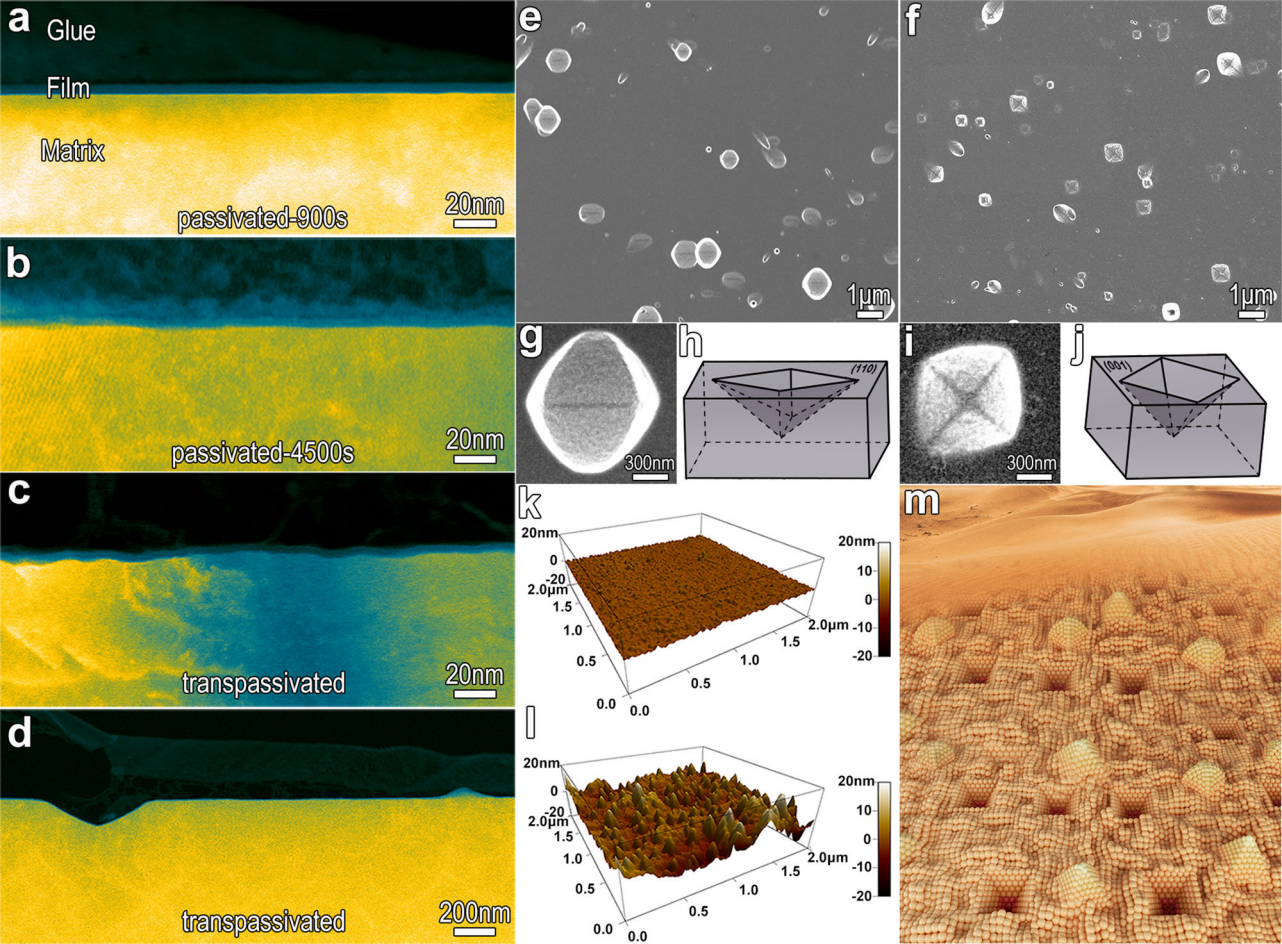
Transpassivation induces undulations at the metal/film interface and roughens the surface of the FeCr15Ni15 alloy. **a**, **b** HAADF-STEM images revealing sharp and straight interfaces of passive films grown in 0.5 mol L^−1^ H_2_SO_4_ electrolyte at 0.4 V for 900 s (passivated-900s) (**a**) and 4500 s (passivated-4500s) (**b**). **c**, **d** HAADF-STEM images showing the undulating interface (with undulation amplitude a few decades of nanometers) for surface films initially grown by passivating in 0.5 mol L^−1^ H_2_SO_4_ electrolyte at 0.4 V/SCE for 900 s, followed by transpassivation at 1.1 V/SCE for 3600 s (transpassivated) (**c**) and the sporadic deeper concaves with depths in the order of a few hundred nanometers (**d**). **e**, **f** Scanning Electron Microscope (SEM) images of the transpassivated surface of the (110) (**e**) and (001) (**f**) plane, showing the pyramidal-shaped deep concaves enclosed by the {111} planes. **g** Zoom-in SEM image of the (110) surface concaves, showing the rhombus appearance dissected by a diagonal line. **h** Schematic illustrating the geometry of the deep concaves formed on the (110) plane. **i** Zoom-in SEM image of the concave on the (001) surface, showing the rhombus appearance comprising of the four triangles. **j** Schematic depicting the geometry of the deep concave formed on the (001) plane. **k**, **l** Atomic force microscope (AFM) images of the passivated (**k**) and transpassivated (**l**) surfaces confirm that transpassivation roughens the surface. **m** Schematic map illustrating the configuration of the TF/Me interface of the transpassivated FeCr15Ni15 alloy. The interface, including the deeper concaves and convexes as well as shallow cones are all enclosed by various {111} planes of the metal matrix.

**Fig. 3:**
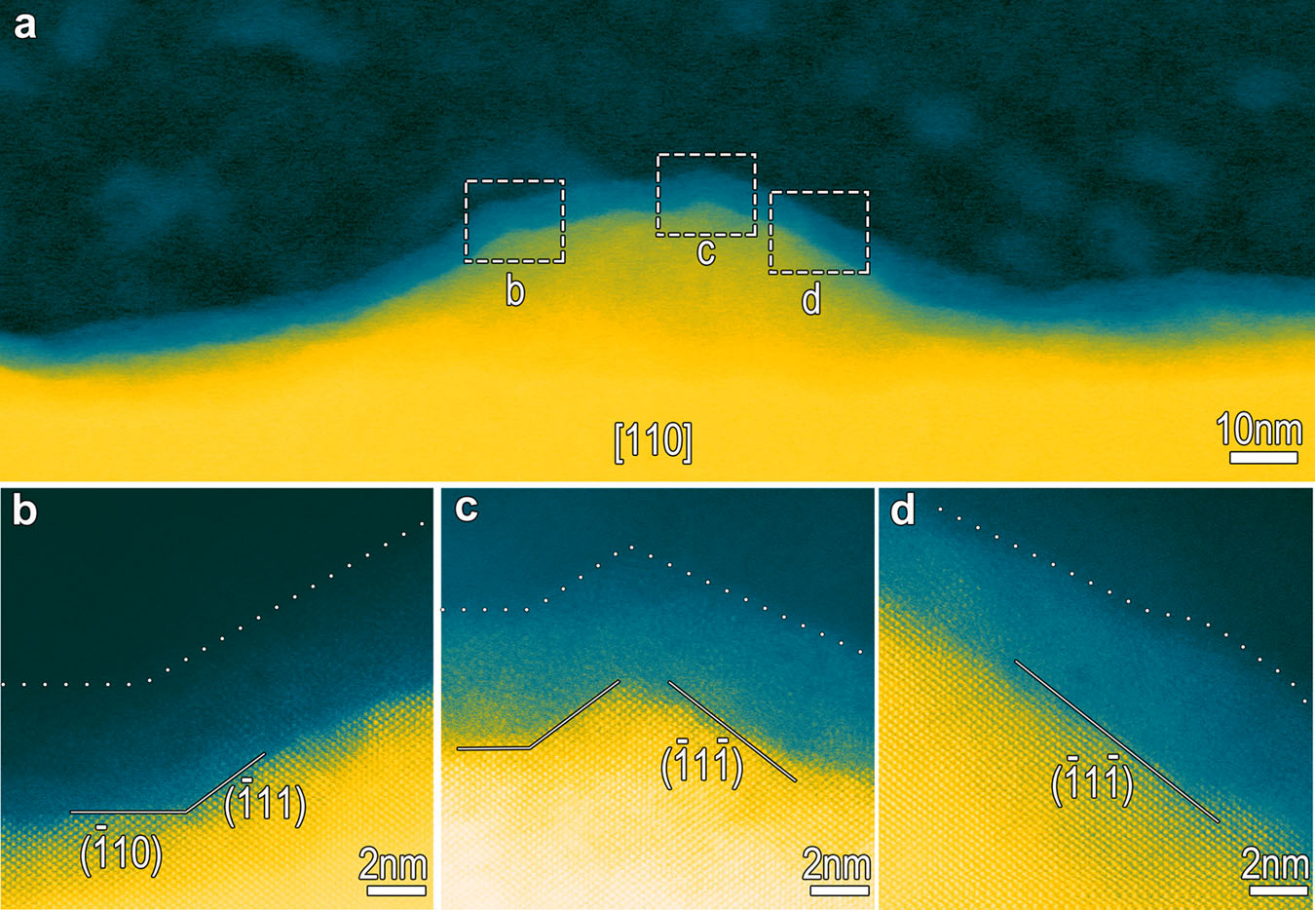
HRHAADF-STEM images showing transpassivation-induced prevalence of the interfaces composed of {111} close-packed planes. **a** HAADF-STEM image showing a convex position. **b**–**d** Zoom-in images show the high-resolution HAADF-STEM images of the three sites located at the top (**b**, **c**) and wall (**d**) of the convex, wherein some specified {111} close-packed planes are exposed.

We apply an extremely positive potential of 1.1 V/SCE, within the transpassive region (as shown in Supplementary Fig. 1), to the passivated FeCr15Ni15 single-crystal alloy, and successfully obtained a fluctuating Me/F interface comprised predominantly of the close-packed {111} planes of the metal matrix, as shown in Figs. 2, 3. In order to confirm the anticipated interface modification, the pristine passive films formed in sulfuric acid are also analyzed. Three types of samples are prepared for the experiments (Supplementary Note 2): (1) Samples passivated in 0.5 mol L^−1^ H_2_SO_4_ at 0.4 V/SCE for 900 s (passivated-900 s); (2) samples passivated in 0.5 mol L^−1^ H_2_SO_4_ at 0.4 V/SCE for 4500 s (passivated-4500 s), and (3) samples initially passivated in 0.5 mol L^−1^ H_2_SO_4_ at 0.4 V/SCE for 900 s and then transpassivated at 1.1 V/SCE for 3600 s (transpassivated). All the films are formed on the (110) planes of the FeCr15Ni15 single crystal. Specimens for cross-sectional transmission electron microscopic (TEM) observation were prepared by bonding passivated or transpassivated surfaces of two samples face-to-face and then thinning by grinding and ion-milling (Supplementary Note 3).

**Fig. 4:**
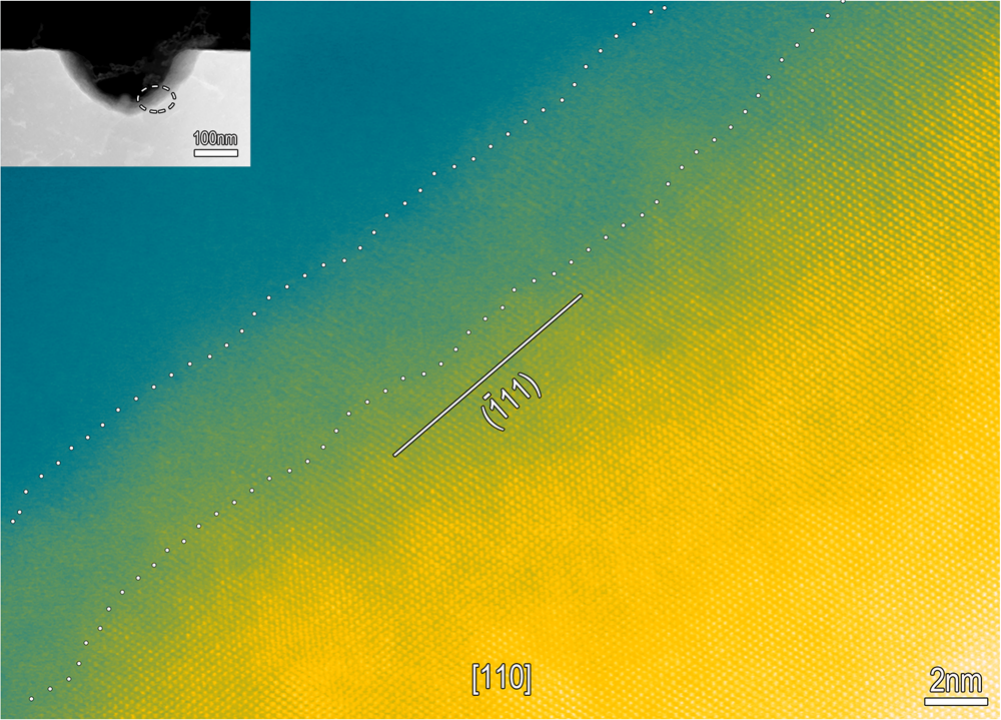
Transpassivation induces the concave wall along close-packed plane. High-resolution HAADF-STEM image at the concave wall along the [110] axis of the FeCr15Ni15 matrix, showing the transpassive film/matrix interface along the (−111) close-packed plane of the austenitic matrix, also in addition to the exposed close-packed plane of the highly-crystalline transpassive film. The transpassive film is found to preferentially grow epitaxially on the single-crystal matrix, with the exposed surface parallel to the interface at nanometer and even atomic scale. Insert shows a deeper concave, where the labeled zone with a dashed line is zoomed-in.

The HAADF-STEM images of the three samples reveal that the Me/F interfaces without transpassivation (samples 1 and 2) are sharp, well-defined and straight (Fig. 2a, b), whereas the metal/transpassive film (Me/TF) interface is substantially undulating (Fig. 2c), with undulation amplitude in the range of a few tens of nanometers. In addition, some sporadic deeper concaves, with triangle-like appearance are observed, with depths in the order of a few hundred nanometers (Fig. 2d). HAADF-STEM imaging of the convex positions within the undulating interface reveals some mini-fluctuations with an amplitude of a few nanometers existing therein (Fig. 3a). Two mini-bulges at the top (labeled with circles b and c) and a wall (labeled with circle d) of the convex are zoomed-in, as shown in Fig. 3b–d. The inclines, both long and short, are all along a specified crystallographic orientation of the austenitic matrix, comprising of the close-packed {111} planes. Similarly, at the wall of the deeper concaves, the Me/TF interface is also along the (−111) close-packed plane of the austenitic matrix (Fig. 4). The outermost surface of the transpassive film is also undulating, keeping step with the interface, while uniformly and continuously covering the matrix (Figs. 2c, 3, 4). Scanning electron microscopic (SEM) (Fig. 2e–j) and atomic force microscopic (AFM) (Fig. 2k, l) imaging of the surfaces of the passivated and transpassivated specimens show that transpassivation treatment actually roughens the surface, forming a large number of pyramid-shaped deeper concaves, enclosed by the {111} planes and having depths of a few hundreds of nanometers (Supplementary Fig. 4). These are interspersed among high-density pyramid-shaped shallow cones, having heights of a few decades of nanometers. The density of the deeper concaves gets to 10^7^/cm^2^ and the cones to 10^10^/cm^2^. Evidently, these high-density concaves and cones are all enclosed by various {111} planes of the metal matrix and covered by the transpassive film, as shown in the schematic on Fig. 2m.

### Concomitant evolution of passive film along {111} plane architecture

The characterized interface comprising of {111} planes are constructed by anodic polarization under a transpassivation potential of 1.1 V/SCE. Previous investigations revealed that transpassivation could often induce significant changes in the chemistry^22–24^, thickness^25^, and compactness^26,27^ of passive films. We thus proceed to identify other possible transpassivation-induced modifications of passive film properties that go hand in hand with the evolution of the {111} plane at the interface.

**Fig. 5:**
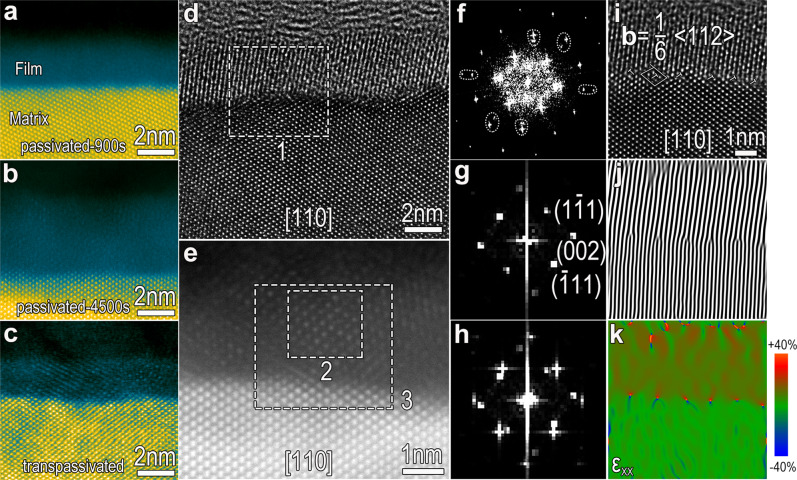
Transpassivation-induced modifications of the passive film. **a**–**c** Transpassivation-induced crystallinity on the surface film. High-resolution HAADF-STEM images along the [110] axis of the austenitic matrix showing the passive films anodically formed in 0.5 mol L^−1^ H_2_SO_4_ electrolyte (**a**, **b**) are almost amorphous with some crystalline constituents, whereas the transpassive film (**c**) is highly crystalline. The atomic columns in the transpassive film are distinct. **d**–**h** Highly-crystalline transpassive film grows epitaxially on the single-crystal matrix. HRTEM image (**d**) and high-resolution HAADF-STEM image (**e**), along the [110] axis of the austenitic matrix, showing the distinct lattice fringes and atom columns in the transpassive films. Fast Fourier transform (FFT) images corresponding to region 1, including the film and matrix (**f**), region 2 including transpassive film (**g**), and region 3 (**h**) illustrate that both phases have a well-defined epitaxial orientation relationship. The lattice parameter of the fcc-structured transpassive film is determined to be 0.43 nm, which is a misfit for the FeCr15Ni15 austenitic matrix with a lattice parameter of 0.36 nm. **i** HRTEM image taken along the [110]_matrix_ direction showing a large number of misfit dislocations with Burgers vector of **b** = 1/6 < 112> at the interface of metal/transpassive film (Me/TF). **j** IFFT (Inverse fast Fourier transform) image of **i** based on the (002) plane showing a periodic array of dislocations along the interface. **k** Geometric phase analysis (GPA) on the Me/TF interface shows enormous strain at the dislocation core.

Cross-sectional high-resolution HAADF-STEM (HRHAADF-STEM) images, obtained along the [110] axis of the austenitic matrix, show the passive films to be mostly amorphous (Fig. 5a, b), and the transpassive film to be highly crystalline (Fig. 5c). Precise analysis by HRTEM and HRHAADF-STEM imaging in combination with fast Fourier transformation (FFT) reveal that the highly-crystalline transpassive film has an fcc structure and lattice parameter of 0.43 nm, having an almost epitaxial orientation relationship with the FeCr15Ni15 austenitic matrix (lattice parameter of 0.36 nm) (Fig. 5d–h). The lattice misfit between the two phases induces a large number of misfit dislocations at the Me/TF interface with Burgers vector of **b** = 1/6 < 112 > (Fig. 5i, j). It is noteworthy that transpassivation at 1.1 V/SCE did not induce film thickening (Fig. 5a–c), thus implying that the improvements in reductive dissolution resistance has nothing to do with increased film thickness, which is indeed contrary to long-held beliefs from earlier reports^28^.

**Fig. 6:**
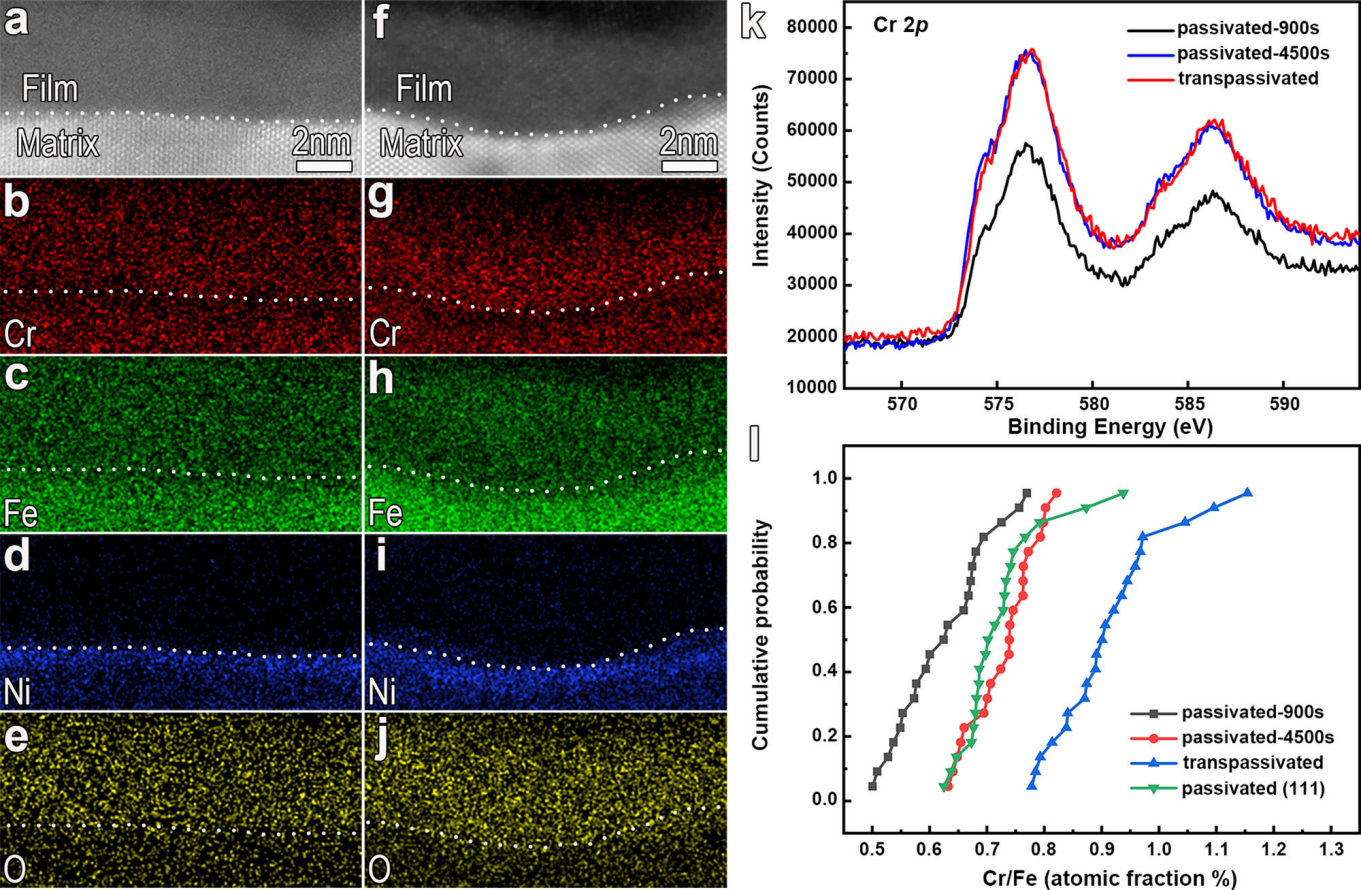
Super-X EDS mapping in TEM and XPS analysis showing that transpassivation at 1.1 V/SCE induces an enhanced Cr-enrichment, while Cr valence is identical in the transpassive and passive films. **a**–**e** High-resolution HAADF-STEM image of the passive film (**a**) and the corresponding elemental maps (**b**–**e**). **f**–**j** High-resolution HAADF-STEM image of the transpassive film (**f**) and the corresponding elemental maps (**g**–**j**). The passive film was formed by potentiostatically polarizing at 0.4 V/SCE in 0.5 mol L^−1^ H_2_SO_4_ for 900 s; while transpassivation was accomplished subsequently at 1.1 V/SCE for 3600 s. **k** XPS analysis showing Cr 2p spectral lines of the three types of films. It is obvious that transpassivation does not induce Cr 2p peak displacement. **l** The cumulative probability analysis on Cr/Fe ratio confirms the enhanced enrichment of Cr induced by the transpassivation treatment.

Super-X EDS mapping analysis does not show pronounced variations in elemental composition and distribution in the transpassive and passive films (Fig. 6a–j). The quantitative analysis of the line-distribution of the compositional elements across the film, as shown in Fig. 6l and Supplementary Fig. 5, indicates that the transpassivation treatment at 1.1 V/SCE enhances the enrichment of Cr in the inner layer of the film rather than transforms the film into a Fe-rich one as reported in some previous work^25^. In another word, at this transpassive potential, preferential dissolution of Fe is enhanced while Cr is enriched in the film, which is consistent with the finding based on the in-operando synchrotron technique in the recent publication^29^. By means of X-ray photoelectron spectroscopy (XPS) analysis, we find that the Cr valence in the transpassive film does not deviate from that in the passive film (Fig. 6k). The transpassivation at the low transpassive potential of 1.1 V/SCE is more like a new-film-growth process as described in the point defect model (PDM)^30,31^ and mixed-conduction model (MCM)^32–36^ rather than a depassivation or film breakdown process. In this case, the evolved interface reactions at the Me/F interface become crystallography-dependent. At the matrix side, especially beneath the deeper concaves, some nanometer-scale zones with darker-contrast are observed, as shown in Supplementary Fig. 6a, b. The darker-contrast could result from the loss of some metal atoms, since image contrast in HAADF mode is approximately proportional to the square of atomic number. Line intensity profile analysis (Supplementary Fig. 6c–e) on the three dashed regions in Supplementary Fig. 6b (labeled by rectangles 1, 2, and 3) reveals that the atom columns with darker-contrast do have lower intensity, further confirming a loss of some metal atoms in those columns. This implies that a large number of metallic vacancies are created at the metal side of the interface.

Evidently, alongside the creation of a large number of close-packed {111} planes at the interface, transpassivating at 1.1 V/SCE also modifies the interface zones via crystallization and enhanced enrichment of Cr on the film side, the introduction of misfit dislocations at the interface, and creation of metallic vacancies at the metal side. Such a series of events were rarely envisaged in the existing theories describing transpassivation-induced evolution in passive films.

### Enhanced corrosion resistance by atomic-scale interface engineering

**Fig. 7:**
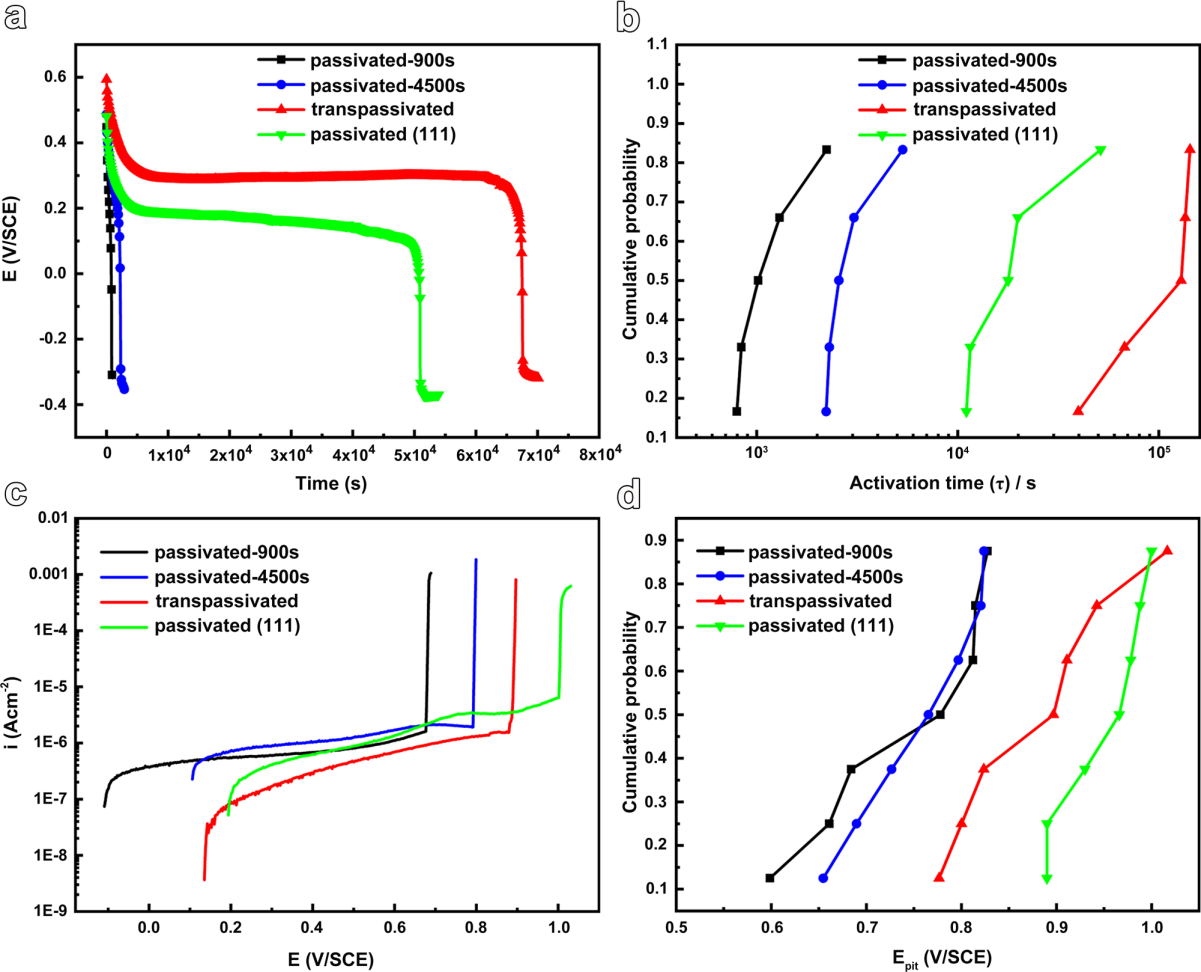
Evaluating the enhancement of the resistance to reductive dissolution and to pitting corrosion induced by the lowest-energy {111} interface. **a** Typical potential decay curves of the four types of samples in 5.6 mol L^−1^ H_2_SO_4_ electrolyte at room temperature show that transpassivation treatment on the non-{111} surface, as well as passivation on the {111}surface, greatly extends the activation time. **b** The cumulative probability analysis of activation time (τ) shows the greatly improved resistance to reductive dissolution of the films on the FeCr15Ni15 single-crystal alloy, induced by transpassivation treatment on the non-{111} surface, as well as passivation on the {111} surface. The τ values were obtained from the potential decay tests in 5.6 mol L^−1^ H_2_SO_4_ electrolyte at room temperature. **c** Typical potentiodynamic polarization curves of the four types of samples in 3.5% NaCl electrolyte at 50 °C. **d** The cumulative probability analysis of pitting potential (E_pit_) shows the enhanced resistance to pitting corrosion induced by transpassivation treatment on the non-{111} surface, as well as passivation on the {111} surface. The E_pit_ values were obtained from the potentiodynamic polarization tests in 3.5% NaCl electrolyte at 50 °C.

The corrosion properties of the FeCr15Ni15 samples that underwent three different passivation treatments as described above are evaluated through potential decay tests in acid solution as well as cumulative probability analysis of the activation time (τ) and pitting potential (E_pit_) in a chloride-containing electrolyte (Fig. 7 and Supplementary Note 4). It is immediately evident that the interface modification remarkably expanded the activation time (τ) of the passive film in sulfuric acid by up to two orders of magnitude (corresponding to the abrupt potential decay) (Fig. 7a, b), and shifts the E_pit_ to more noble values by about 120 mV on average (Fig. 7c, d). It is noteworthy that the contribution of the close-packed {111} planes on the improved resistance to reductive dissolution as well as to pitting corrosion is mixed into the possible positive effect of the higher Cr/Fe ratio in the film after transpassivation treatment, since usually Cr/Fe ratio is considered to be one of the parameters indicating the quality of passive films. To elucidate the significant role of the low-energy {111} planes, the FeCr15Ni15 single crystal is cut to expose a {111} surface and then passivated (passivated (111)), whose corrosion resistance is examined. It is indicated that passivation on the {111} surface indeed exhibits an exceptional corrosion resistance compared with the counterpart of non-{111} surface (Fig. 7), thus indicating that the close-packed {111} planes greatly improve the resistance to reductive dissolution as well as the pitting corrosion of the films on the FeCr15Ni15 single-crystal alloy since the passive films on the (111) and (110) share the similar Cr/Fe ratio (Fig. 6l and Supplementary Fig. 5m) and microstructure (Supplementary Fig. 7).

In order to rationalize that the experimentally obtained expanded activation time (τ) by up to two orders of magnitude is mainly contributed by the {111} facets at the interface, we have performed first-principle calculations to model the ratio of the surface energy and surface atom density modulating the crystallographic plane-dependent corrosion behavior. Details of the computational procedure can be found in Supplementary Note 5. The obtained results (Supplementary Fig. 8) show that the ionization rate along [110] is dozens of times faster than that along [111] direction. Evidently, the expansion magnitude in the activation time (Supplementary Note 4 and Fig. 7a, b) is equivalent to the differential limit derived by the crystallography-dependent surface energy density.

### Distribution of Cl in the transpassive film

**Fig. 8:**
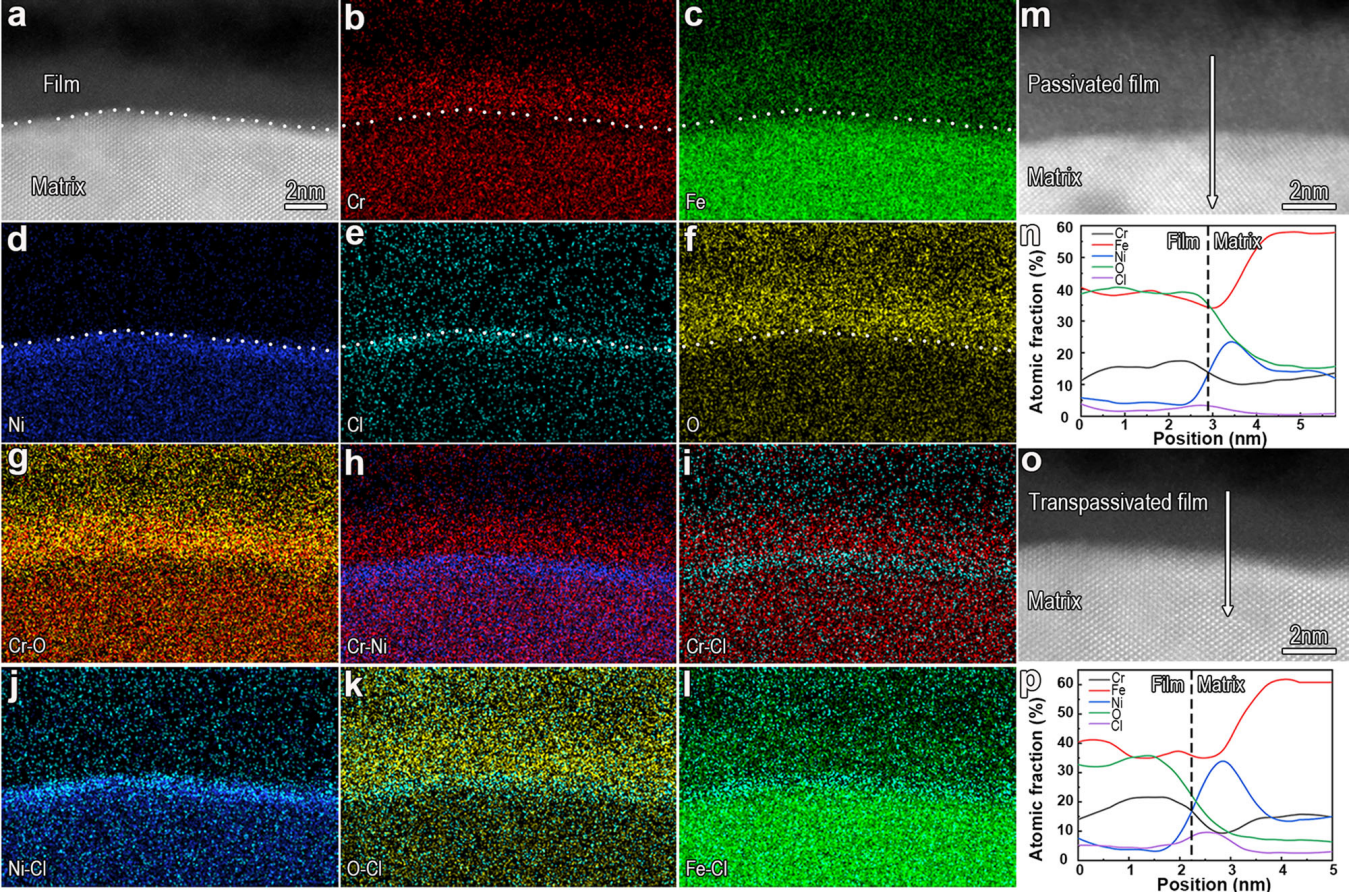
Chloride ions incorporated in and penetrating the transpassive film, as well as accumulating at the metal side of the matrix/transpassive film (Me/TF) interface. **a** HRHAADF-STEM image along the [110]_matrix_ direction showing a sharp Me/TF interface. The transpassive film was immersed in 0.5 mol L^−1^ H_2_SO_4_ + 0.3 mol L^−1^ NaCl electrolyte at room temperature for 24 h. **b**–**l** Element maps (**b**–**f**) and composite maps (**g**–**l**) clearly reveal that chloride ions accumulate underneath the transpassive film. The dotted line specifies the Me/TF interface. **m** High-resolution HAADF-STEM image along the [001]_matrix_ direction showing a sharp Me/F interface. The passive film was immersed in 0.3 mol L^−1^ NaCl + 0.5 mol L^−1^ H_2_SO_4_ electrolyte at room temperature for 24 h. **n** Line distribution of elements across the passive film/metal (Me/F) interface along the white arrowed line in (**m**). **o** High-resolution HAADF-STEM image along the [110]_matrix_ direction showing a sharp Me/TF interface. The transpassive film was immersed in 0.5 mol L^−1^ H_2_SO_4_ + 0.3 mol L^−1^NaCl electrolyte at room temperature for 24 h. **p** Line distribution of elements across the TF/Me interface along the white arrowed line in (**o**).

The transpassivated FeCr15Ni15 alloy was immersed in a chloride-containing electrolyte at room temperature for 24 h and then subjected to Super-X EDS mapping experiments. We obtain the distribution of elemental Cl in the transpassive film, as shown in Fig. 8, which shows that Cl is not concentrated within the inner layer like that of the passive film elucidated in our previous work^37^, but rather accumulates at the metal side, immediately beneath the transpassive film. We further confirmed the precision of our observed locations of Cl at an extremely narrow interface region with a width of only 1~2 nanometers by superimposing two maps to yield composite images, as shown in Fig. 8g–l. It is shown that the strip predominantly occupied by Cl just overlaps the Ni-enriched strip (Fig. 8j), staggers the Cr-rich strip (Fig. 8i), and is immediately adjacent to the O-strip (Fig. 8k). The relative position of Cl to the other elements indicates that Cl is indeed located at the metal side. In addition, the distribution of Cl density across the film is demonstrated by digitizing the EDS maps, as shown in Fig. 8m–p. It also clearly shows Cl concentration within the inner layer of the passive film (Fig. 8m, n) and Cl accumulation at the matrix side of the transpassive film (Fig. 8o, p).

## Discussion

In the present work, we apply transpassivation treatment at 1.1 V/SCE to accomplish atomic-scale modification to the Me/F interface and also to distinguish the concomitant structural evolutions in the passive films, allowing a re-evaluation of the atomic-scale mechanism of transpassivity, which remains one of the unresolved fundamental issues in corrosion science. Transpassivation occurs when the applied potential becomes too positive and is characterized by enhanced anodic current densities^22,38–42^, as shown in Supplementary Fig. 1a. The high anodic current is usually treated as an indication of a rapid dissolution of a once-passivated metal surface. Transpassivation, accordingly, is often referred to as transpassive dissolution^25^, thus creating the long and deeply held impression that transpassivation brings damage. It is generally believed that transpassivation processes in Fe-Cr alloys and stainless steels involve conversion or transformation of some chemical species in the original passive film into higher-valance or more soluble products^22,24,25,40,41,43–51^. Expectedly, a number of hypotheses and models have been proposed to describe the detailed electrode reactions, based on detection of the transpassivation-induced evolution in chemistry and elemental valence of the passive film by means of surface spectroscopies (XPS, AES, XANES)^24,52–54^, as well as the detection of possible intermediate products using electrochemical methods (CV, RRED, EIS)^22,25,40,46–51^. A general consensus is that trivalent chromium in the passive film is oxidized to hexavalent chromium species, which enter into solution^22,24,25,40,41,43–47,49–51,53,54^. This brings about significant changes in the chemistry of the passive film, which transforms from a Cr-rich passive film into a Fe-rich transpassive film^25^. In other words, hexavalent chromium might be involved in the transpassivation, but it does not necessarily exist in the film. Meanwhile, transpassivation was as well thought to induce film thickening^23,25,26,28,53,55–58^, becoming porous^26,27^ and lowering of electrical resistance, leading to a remarkable reduction of corrosion resistance^27,53,54,59^. Thus, transpassivation was arbitrarily concluded to be a damaging process and deteriorate the corrosion resistance based on the dramatic enhancement of anodic current densities at the transpassivation potential region rather than on the basis of systemic examinations of the resultant structural evolution as well as the corrosion resistance at open circuit potential; also the consensus on chromium oxidation was simply reached without sufficient insights on the possible mechanisms of the oxidative dissolution of Cr, which has since remained a controversy. Most investigators declare that the oxidative dissolution occurs at oxide film/solution (F/S) interface^42,60–62^, whereas some insist, on the basis of XANES results, that there is no dissolution of chromium oxide in the passive film during transpassivation^63^. Recently, Langberg et al.^29^, based on the operando synchrotron technique, reports that different processes are involved with varied potentials within the transpassive potential range. At the relatively lower transpassivation potentials, the preferential dissolution of Fe occurs; whereas the rapid dissolution of Cr occurs at much higher transpassive potentials. In this sense, it is reasonable that the transpassive film formed at 1.1 V/SCE is enriched of Cr^3+^ and free of hexavalent chromium. Another school of thought holds that the alloy elements in the metal matrix could be directly dissolved across the oxide film into solution^26,42^.

Our observed interface roughening, coupled with the proliferation of exposed {111} planes strongly implies that the transpassivation at 1.1 V/SCE specifically induces metal matrix dissolution. At potentials within the passive region, it is difficult to pull out the metal atoms from the lattice into solution, across the compact passive film, hence the metal matrix is only very slightly dissolved. Conversely, when a potential shifts into the transpassive region, the prevailing conditions and considerable driving force can remarkably enhance the direct dissolution of the metal matrix across the oxide film into the solution. The anodic dissolution of Fe is greatly accelerated in such situations, wherein the atoms readily escape from lattice positions along the <110> direction, with the rapid dissolution exposing the {111} residual plane at the interface, as illustrated in Supplementary Fig. 9.

The non-homogeneous dissolution along different orientations creates undulations at the interface. The depths of the various concaves vary substantially, with a few that are quite deep. The convex regions also exhibit some nanometer-scale fluctuations. All of these give the impression that the dissolution initiation has a temporal order. On the basis of a series of modifications in the films and at the Me/F interfaces, we propose a continuous process to rationalize the above characteristics of the undulating interface. In the original amorphous passive film, some nano-crystals at the interface introduce misfit dislocations (Supplementary Fig. 10). GPA (Geometric Phase Analysis) analysis reveals considerable strains at the dislocation core (Fig. 5k), which induces an electrode potential drop^64^, being a preferential dissolution site. On application of the transpassivation potential of 1.1 V/SCE, the preferential crystallography-dependent dissolution of the metal matrix would initiate at these dislocation sites and then propagate into deep concaves. With transpassivation, the pristine amorphous-dominated passive film becomes more epitaxially crystallized, thereby introducing a large number of misfit dislocations at the interface (Fig. 5i). These dislocations, as new initiation sites, then would trigger new dissolution and subsequent propagation. The concaves with different depths would be connected with propagation. The outcome is a fractured interface with large undulations and embedded mini-fluctuations comprised of {111} crystallographic planes. It is noteworthy that the alternative contributor to the formation of various depths of concaves might be the inhomogeneous composition distribution in the films. The planar inhomogeneity of Cr-enrichment in the passive film at the nanoscale has been determined based on detecting the local electrical resistance by conductive AFM^65^. The location enriched in chromium oxide would be more resistant and thus block the local dissolution.

As to the chloride attack on the oxide film, our earlier work^37^ has unmasked the attack mechanism and identified chloride transportation across the film, interfacial undulations, and lattice-induced tensions as the primary processes. The undulations result from a local accumulation of chloride at the interface and consecutive interactions with the metal. Therefore, inhibition of any involved step would be expected to effectively improve pitting resistance. In chloride-containing media, two consecutive processes act together to hinder chloride accumulation at the Me/F interface as well as weaken the interactions between chloride and metal. Firstly, the {111} planes with low surface energy would weaken the interaction with chloride; a large number of dislocations would facilitate transport and dispersal of chloride at the interface, which hinders interfacial accumulation and distribution of chloride. Secondly, a large number of metal vacancies (as shown in Supplementary Fig. 6) produced by the remarkably enhanced metal side dissolution would act as sinks to capture chloride, further lowering chloride accumulation at the interface. Indeed, we have examined the accumulation of chloride at the metal side immediately beneath the transpassive film (Fig. 8). Evidently, all the events evolved in the modifications to the film by the transpassivation treatment, including the film crystallization, exposure of close-packed planes, formation of misfit dislocations at the interface, as well as creation of metal vacancies at the metal side, can effectively weaken the impact of chloride ions on the metal matrix and thus improve the pitting resistance of the FeCr15Ni15 austenitic alloy. In all, transpassivation at 1.1 V/SCE does actually promote the anodic dissolution of passive metals, but this cannot be arbitrarily treated as corrosion damage. Rather, it is more like a process of survival of the fittest, wherein active atoms are sacrificially dissolved, while relatively inactive atoms remain more or less intact, giving rise to a barrier interface comprised of the close-packed {111} planes. Clearly, the so-called stability of the surface film depends on factors like the nature of the film itself, the nature of the interface, and the structural features directly underlying the metal matrix. Indeed, the strategy of atomic-scale interface engineering, achieved by transpassivation, has broad prospects for useful practical applications in anti-corrosion interventions.

To examine the effectiveness of the transpassivation approach on other metals, we have performed the transpassivation treatment on a commercial 304 austenitic stainless steel (304 SS). In contrast to the single-crystal alloy, commercial stainless steels involve grain boundaries (GBs) and inclusions. In order to examine the role of GBs and inclusions in engineering low-energy interface on the metal side, two kinds of samples with smaller (2 mm × 2 mm) and larger area (10 mm×10 mm) were prepared (Supplementary Fig. 11 and Supplementary Note 6). The smaller samples have little probability to expose inclusions, which is suitable for investigating the effect of GBs on the interface engineering by transpassivation; the larger samples are applied to examine the effect of inclusions.

**Fig. 9:**
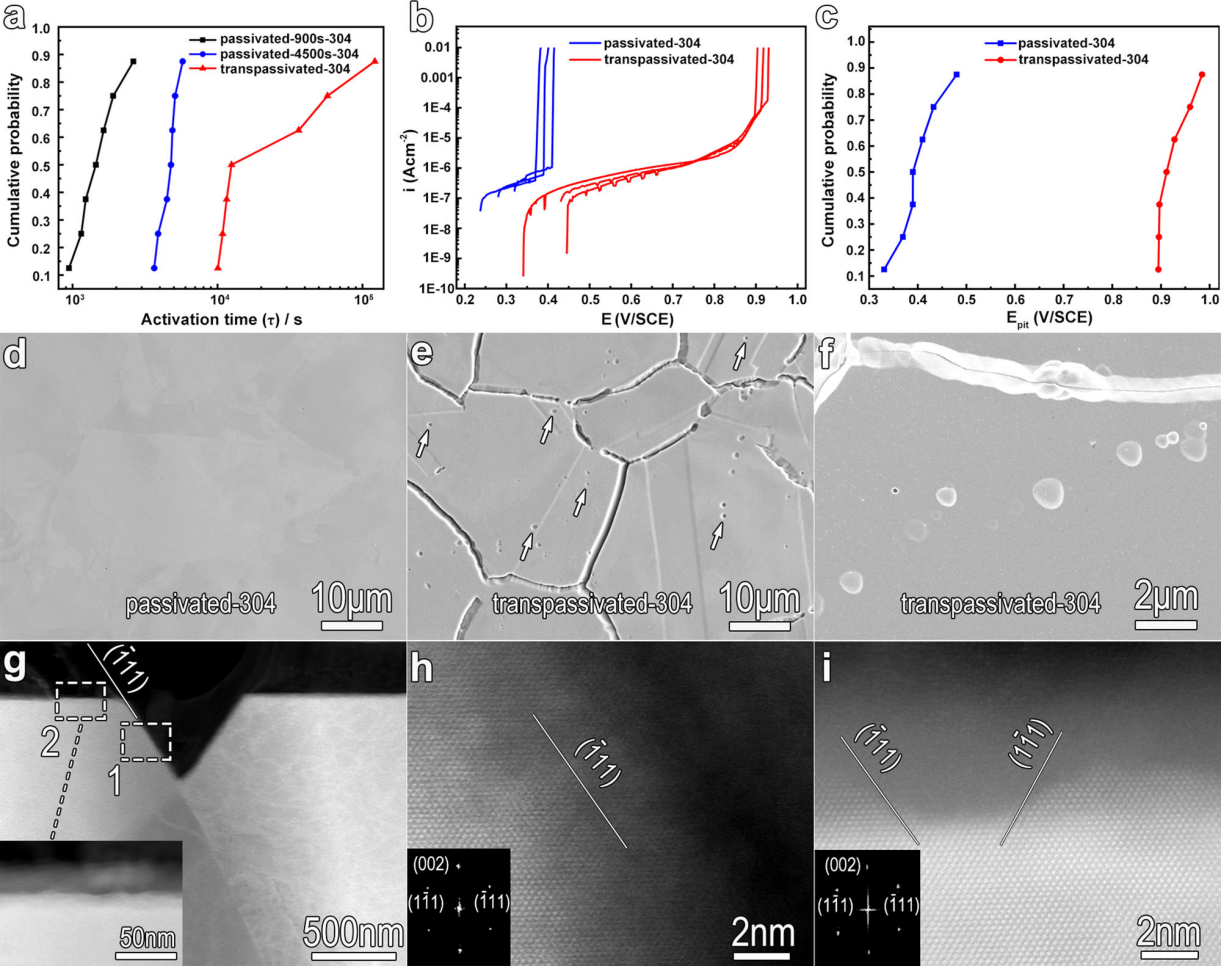
The transpassivation strategy applied to the commercial stainless steel. **a** The cumulative probability analysis of activation time (τ) shows the improved resistance to reductive dissolution of the films on the 304 SS with small exposure areas (2 mm × 2 mm), induced by transpassivation treatment. The τ values were obtained from the potential decay tests in 5.6 mol L^−1^ H_2_SO_4_ electrolyte at room temperature. **b** Typical potentiodynamic polarization curves, in 0.5 mol L^−1^ H_2_SO_4_ + 3.5% NaCl electrolyte at 50 °C, of the 304 SS. Before potentiodynamic polarization measurement, some of the 304 SS samples were potentiostatically passivated at 0.4 V/SCE in 0.5 mol/L H_2_SO_4_ electrolytes for 4500 s (passivated-304), and the others were initially passivated in 0.5 mol L^−1^ H_2_SO_4_ at 0.4 V/SCE for 900 s and then transpassivated at 1.1 V/SCE for 3600 s (transpassivated-304). **c** The cumulative probability analysis of pitting potential (E_pit_) shows the greatly enhanced pitting resistance of the 304 SS induced by transpassivation treatment. **d** SEM image showing the smooth surface of the electrochemically polished 304 SS sample after passivation in 0.5 mol L^−1^ H_2_SO_4_ solution at 0.4 V/SCE for 4500 s. **e** SEM image of the transpassivated surface showing the presence of GBs and dispersal of small pits in the interior of the grains. **f** Zoom-in SEM images of the GBs and the small pits, showing the GBs have gully-like morphology, and the small pits have a regular appearance. **g** HAADF-STEM image showing a typical steep concave with depth in the order of a few hundred nanometers, formed along the GB after transpassivation treatment. Inset is the zoom-in image of the grain surface labeled by the rectangle 2 in **g**, showing some mini-fluctuations. **h** Zoom-in image shows the high-resolution HAADF-STEM image of the rectangular labeled region 1, located at the wall of the concave in **g**. The wall is along the close-packed {111} plane. **i** Zoom-in image shows the high-resolution HAADF-STEM image of the rectangular labeled bump 2 in **g**, wherein some specified {111} close-packed planes are exposed. Insets of panels **h** and **i** show the Fast Fourier Transform (FFT) images of the high-resolution HAADF-STEM image.

For the 304 SS with small exposure areas, we obtain a great improvement in the resistance to the reductive dissolution in acid media, expanding the activation time (τ) of the transpassive film in sulfuric acid by up to two orders of magnitude (Fig. 9a, Supplementary Fig. 12, and Supplementary Note 6). The potentiodynamic polarization experiments indicate that the transpassivation treatment at 1.1 V/SCE obviously decreases the passive current density and shifts the pitting potential of 304 SS to a noble value as large as 520 mV approximately in acidic chloride-containing media (Fig. 9b, c). SEM images of the passivated and transpassivated surfaces show that the transpassivation treatment at 1.1 V/SCE results in enhanced visibility of the grain boundaries (GBs), which indicates that transpassivation dissolution of the metal matrix is preferentially along the GBs (Fig. 9d, e). It is seen that the GBs become transformed into the gully-shaped steep concaves. Meanwhile, some small pits are observed to be dispersed in the interior of the grains (Fig. 9e). From the zoom-in images (Fig. 9f and Supplementary Fig. 13a–c), it is seen that these pits have regular appearances and they share quite similar geometric configurations within a single grain. This implies that, although the orientations differ between grains, the transpassive dissolution of the metal matrix underneath the film proceeds along specific crystallographic orientations leaving the low-energy planes. By AFM imaging of the passivated surface and imaging within a single grain, (excluding the pits) of the transpassivated surface (Supplementary Fig. 13d–f), it can be confirmed that transpassivation treatment roughens the surface, forming a large number of shallow cones, similar to the case of the FeCr15Ni15 single-crystal alloy. The HAADF-STEM images reveal that the steep concaves formed along GBs have a triangle-like appearance and have depths in the order of a few hundred nanometers (Fig. 9g and Supplementary Fig. 14). Based on the high-resolution HAADF-STEM imaging (Fig. 9h and Supplementary Fig. 14b, d), the Me/TF interfaces at the wall of the concaves at the GBs are determined to be either along the {111} plane (Fig. 9g and Supplementary Fig. 14a) or composed of highly dense {111} zigzag facets (Supplementary Fig. 14c). In the case that the wall of the concaves features zigzag, the terminal plane might macroscopically deviate somewhat from the {111} (Supplementary Fig. 14c, d). HAADF-STEM image also shows that the transpassivation induces undulation of the Me/F interface at the interior of grains (inset of Fig. 9g), with undulation amplitude in the range of a few tens of nanometers. High-resolution HAADF-STEM imaging of the mini-fluctuations reveals the close-packed {111} planes (Fig. 9i). Evidently, the transpassivation treatment at 1.1 V/SCE restructures the interface, forming a large number of deeper concaves and a high density of shallow cones at the interior of grains, as well as gully-shaped steep concaves at GBs. These high-density concaves and cones are enclosed by various {111} planes or highly dense {111} zigzag facets of the metal matrix and all of them are covered by the transpassive film. Clearly, the preferential transpassive dissolution at GBs of the commercial stainless steels, which had been considered as corrosion damage, is in fact a reconstruction of the Me/F interface with an emerging of low-energy roughened interface. In other words, grain/grain boundary engineering yields a reconfigured interface, which enhances the stability of the passive film and ultimately improves corrosion resistance. It is worthwhile to note that the enhanced pitting resistance is contributed by the improved resistance to pit initiation since the crystallographic engineering is actually a surface treatment and would not influence the pit growth stability.

For the 304 SS with large exposure areas, it is likely that the transpassivation treatment doesn’t lead to an enhancement of corrosion resistance. By examining the SEM morphologies (Supplementary Fig. 15), we find that the evident local corrosion damage occurs at the coarse sulfide-oxide composite inclusions, while the representative morphology of small pits rarely forms in the interior of the grains (Supplementary Fig. 15a). This implies that coarse inclusions weaken the effect of interface engineering by anodic transpassivation potential. We have also examined a 304 SS (Nippon steel corporation in Japan) with a small size of inclusions (Supplementary Fig. 15b). It is found that the transpassivation rebuilds the GBs into the gully-shaped steep concaves and produces small pits with regular appearances dispersing in the interior of the grains (Supplementary Fig. 15c–f), similar to the results of the small 304 SS samples.

Based on the above observations, it is clearly seen that transpassivation does not always correspond to a damaging process as generally cognized. The existing idea that transpassivation brings damage might result from the fact that the coarse inclusions amplify the effects of local electrochemical corrosion cells and weaken the capability of crystallographic engineering by transpassivation. In this sense, it is worthwhile to emphasize that manufacturing high-purity steels by removing coarse-size inclusions is still one of the major concerns in steel-making processing, since they are not only harmful to the mechanical properties of steels but also debase the efforts in improving the corrosion resistance. We also propose that the low-energy interface engineered by other possible approaches could also enhance the corrosion resistance of stainless steel.

In summary, this study provides a methodology to accomplish great enhancement of corrosion resistance by means of an atomic-scale reengineering of the Me/F interface. Using spherical aberration-corrected transmission electron microscopy, we have directly monitored the dissolution events on the underlying metal matrix during passive film degradation in acid media and found the dissolution to be anisotropic with crystallographic orientation. Accordingly, we conceive a strategy for improving the stability of the passive film, which involves creating an inactive Me/F interface. By subjecting a passivated FeCr15Ni15 single-crystal alloy as well as commercial 304 stainless steel to a certain potential in the transpassive range, we have obtained a fluctuated interface where the metals are modified and bounded by seemingly inert close-packed {111} crystallographic planes. The as-received inactive interface greatly improves the stability of the passive film and makes the metals remarkably resistant to degradation in acid media as well as to chloride attack. We also analyze the concomitant modification of the passive film architecture by the close-packed {111} planes, including induced crystallinity on the film side, the occurrence of misfit dislocations at the interface, and dissolution-induced generation of metallic vacancies at the metal side. By precisely detecting the chloride distribution at the Me/F interface, we find that chloride accumulates at the metal side immediately beneath the transpassive film. Our experimental results suggest that the misfit dislocations facilitate chloride transportation at the interface, whereas the metallic vacancies act like traps that capture the chloride. This effectively weakens the local accumulation of chloride ions at the interface and thus contributes to the enhancement of the pitting resistance.

## Methods

### HAADF-STEM imaging

HAADF-STEM images were recorded using aberration-corrected transmission electron microscopy (Titan Cubed G^2^ 60-300 and Titan Cubed Themis G^2^ 300 microscopes, fitted with a high-brightness field-emission gun (X-FEG), double Cs corrector from CEOS, and a monochromator operating at 300 kV; configured with the fast-speed Super-X EDS detector). The beam convergence is 25 mrad, and thus yields a probe size of <0.1 nm.

### GPA analysis

GPA is an effective approach to determine crystal lattice variations (strains) in a large area of high-resolution TEM or STEM images^66^, and it shows great potential in mapping the strains near the dislocation core. Here the method of GPA is used to extract the strains in the Me/TF (metal/transpassive film) interface. In the GPA method, all lattice changes, or strains obtained from a specific STEM image, were treated as “elastic” in mathematics even if some defects (such as dislocations) are involved, since only lattice parameters were extracted from the STEM image. GPA can be achieved by using the custom plugins of GPA in Gatan Digital Micrograph (DM) software for TEM imaging analysis^67,68^.

### Representativeness of the (S)TEM images

We confirm the representativeness of the images on a very fine-scale in two aspects. One is that we acquire a large number of fine-scale (S)TEM images at various locations in variant specimens, ensuring that the evolution of structural characteristics is representative; another is that we characterize the microstructures at the multi-scales, making TEM observation satisfactory equivalent to the AFM and SEM imaging.

## Supplementary information

Supplementary Information

Peer Review File

## Data Availability

All data supporting the key findings of this study are available within the article and its Supplementary Information. All raw data generated during the current study are available from the corresponding author on reasonable request.
