# Supplementary material for: Enhanced corrosion resistance by engineering crystallography on metals
Source: Nat Commun. 2022 Feb 7;13:726. doi: 10.1038/s41467-022-28368-8 (PMC8821614; doi:10.1038/s41467-022-28368-8)
Supplement: Supplementary file 1 — Supplementary Information [file 41467_2022_28368_MOESM1_ESM.pdf]

Supplementary Information for:

Enhanced corrosion resistance by engineering crystallography on metals

X. X. Wei[1,2]†, B. Zhang[1]†, B. Wu[3], Y. J. Wang[1], X. H. Tian[1,2], L. X. Yang[1], E. E. Oguzie[4], X. L. Ma[1,5]*

[1]Shenyang National Laboratory for Materials Science, Institute of Metal Research, Chinese Academy of Sciences, Wenhua Road 72, Shenyang 110016, China

[2]School of Materials Science and Engineering, University of Science and Technology of China, Shenyang 110016, China

[3]Bay Area Center for Electron Microscopy, Songshan Lake Materials Laboratory, Dongguan, Guangdong 523808, China

[4]Africa Centre of Excellence in Future Energies and Electrochemical Systems, Federal University of Technology Owerri, P.M.B 1526, Owerri. Imo State, Nigeria

[5]State Key Lab of Advanced Processing and Recycling on Non-ferrous Metals, Lanzhou University of Technology, Lanzhou 730050, China

†These authors contributed equally: X. X. Wei, B. Zhang

*Corresponding author. E-mail: xlma@imr.ac.cn

This PDF file includes 15 Supplementary Figures, 6 Supplementary Notes and 4 Supplementary References.

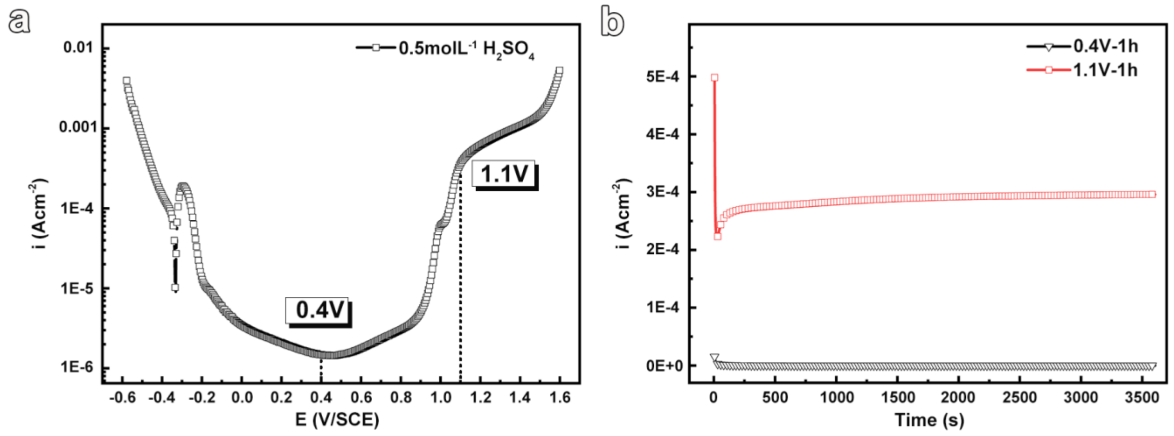

Supplementary Fig. 1. Potentiodynamic polarization curve (a) and potentiostatic polarization curve at a constant potential of 0.4 V (located within the passive region) and 1.1 V (located within the transpassive region) (b) of FeCr15Ni15 in 0.5 mol L^{-1} H_2SO_4 electrolyte.

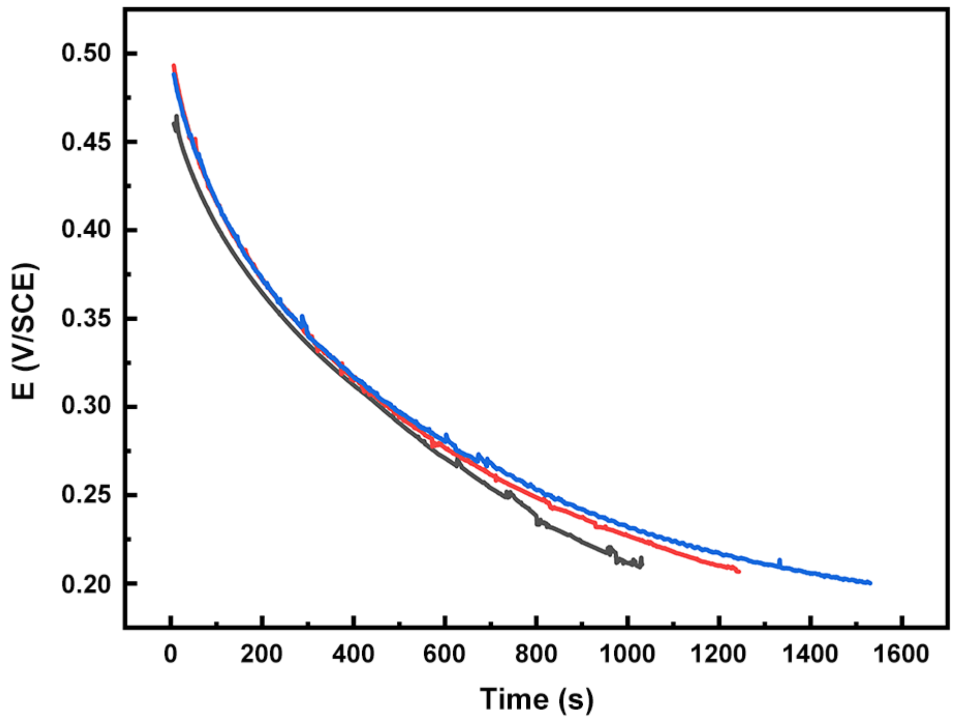

Supplementary Fig. 2. Potential decay curves of passivated FeCr15Ni15 alloy in 5.6 mol L^{-1} H_2SO_4 electrolyte at room temperature. $FeCr_{15}Ni_{15}$ alloy was firstly passivated at 0.4 V/SCE for 900 s in 0.5 mol L^{-1} H_2SO_4 electrolyte. The passivated specimens were subsequently immersed in 5.6 mol L^{-1} H_2SO_4 electrolyte and open circuit potentials (OCPs) were recorded. After about 17~25 min, when the OCP value attained 0.2 V/SCE, the specimens were taken out from the sulphuric acid ready to further TEM observation in order to visualize the possible structural evolution of Me/F interfaces induced by "reductive dissolution".

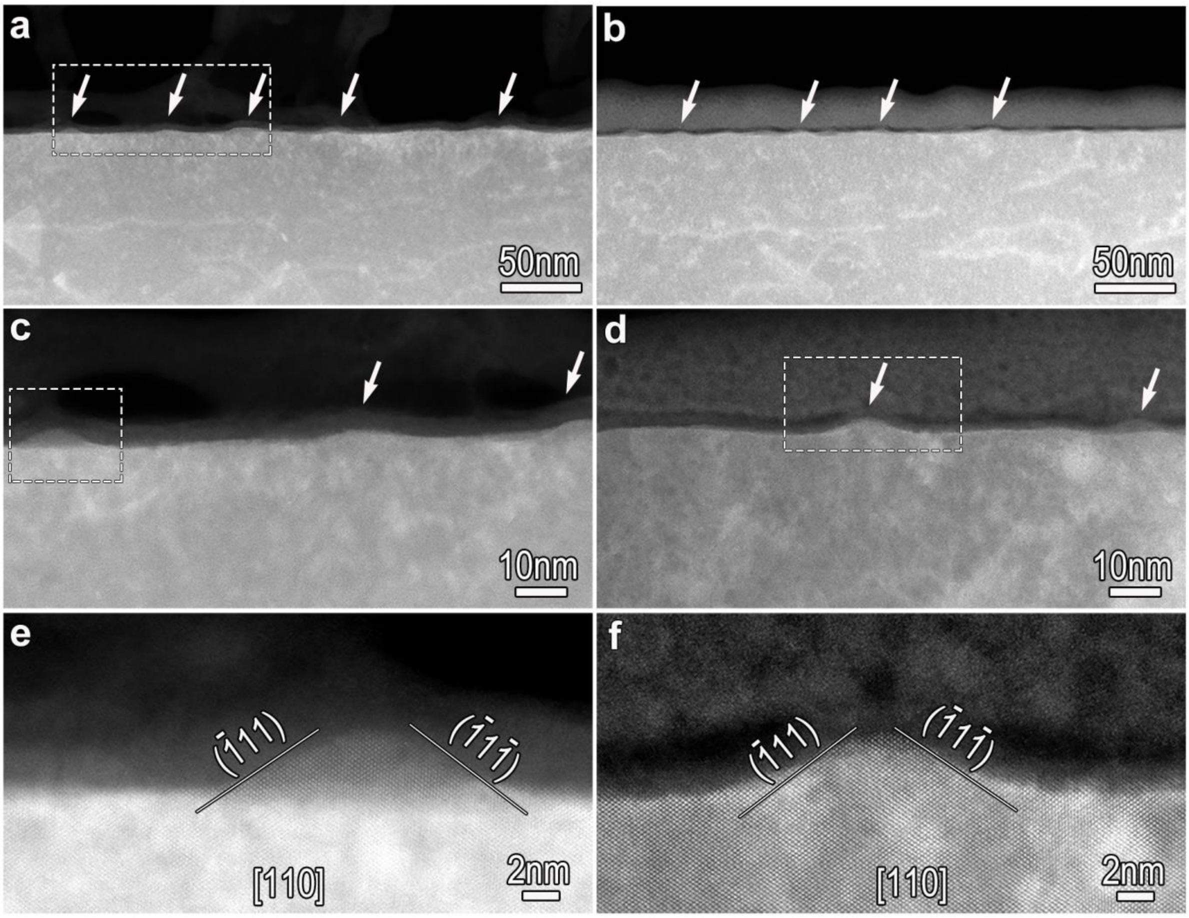

Supplementary Fig. 3. Features of the Me/F interface after immersion in sulphuric acid. (a-d) HAADF-STEM images showing some bumps (labeled with white arrows) at the Me/F interface at varying magnifications. c is the zoom-in image of the rectangular labelled region in a. (e-f) Zoom-in images of the bumps enclosed by dashed rectangles in c and d, showing the walls along the close packed {111} plane.

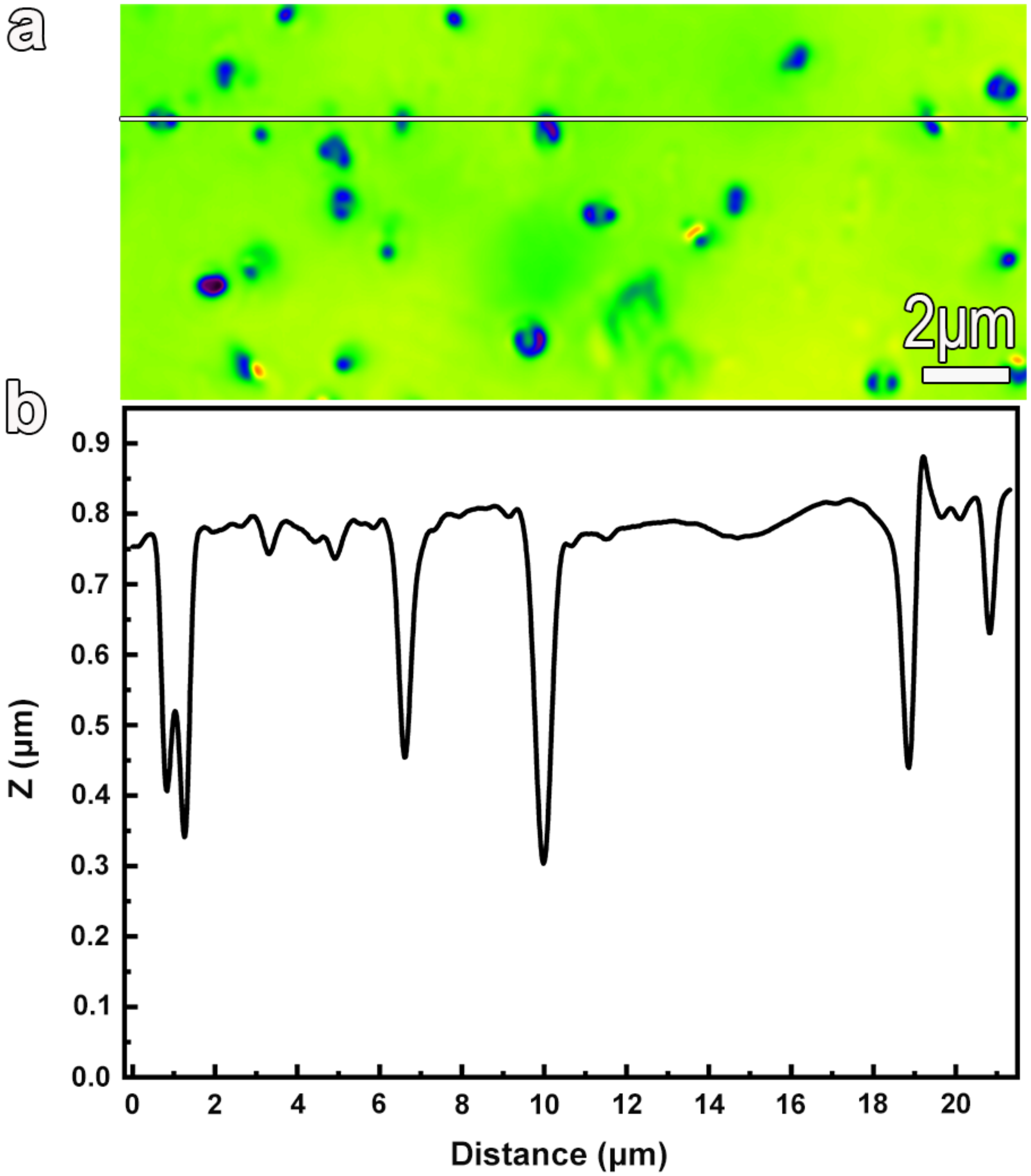

Supplementary Fig. 4. Confocal Laser Scanning Microscopy (CLSM) analysis showing the depths of the deep concaves induced by transpassivation treatment to be 200~400 nm. (a) CLSM image showing the distribution of the deep concaves induced by the transpassivation treatment. (b) The depth analysis of the concaves along the line in a.

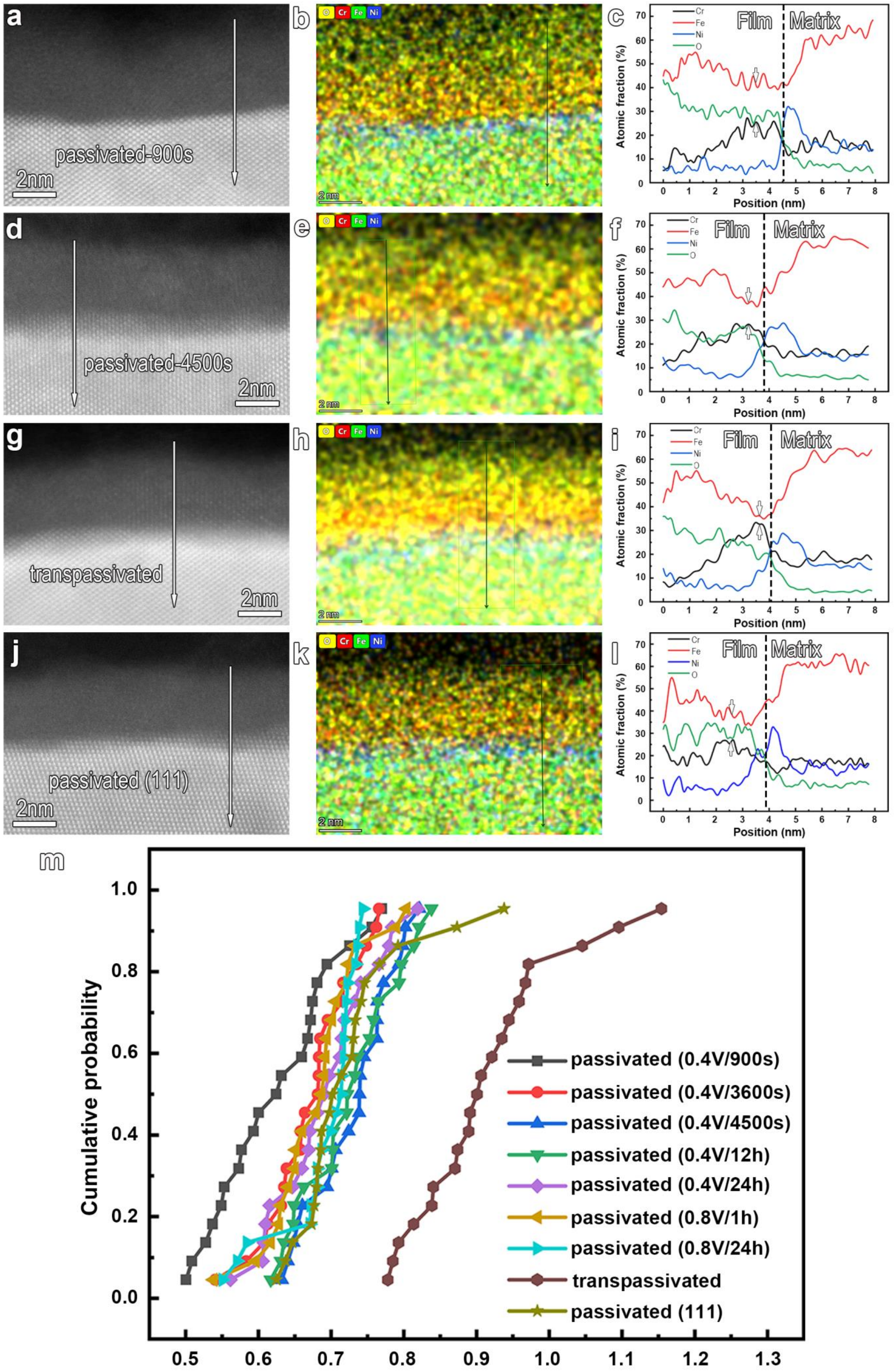

a
passivated-900s
2nm
b
Cr
Fe
Ni
c
Film
Matrix
Atomic fraction (%)
Position (nm)
d
passivated-4500s
2nm
e
f
Film
Matrix
g
transpassivated
2nm
h
i
Film
Matrix
j
passivated (111)
2nm
k
l
Film
Matrix
m
Cumulative probability
passivated (0.4V/900s)
passivated (0.4V/3600s)
passivated (0.4V/4500s)
passivated (0.4V/12h)
passivated (0.4V/24h)
passivated (0.8V/1h)
passivated (0.8V/24h)
transpassivated
passivated (111)

Supplementary Fig. 5. Quantitative analysis illustrating the line-distribution of the compositional elements across the film. (a) High resolution HAADF STEM images showing the sharp and well defined Me/F interfaces of the FeCr15Ni15 alloy after passivation at 0.4 V/SCE for 900 s. (b) The EDS map covering the view field of the image a, in which the information on the line-distribution of elements is extracted along the vertical white arrowed line. (c) Line distribution of the elements of Fe, Cr, Ni and O across the film in a. (d-1) Analysis of the line-distribution of elements on the films corresponding to the other three conditions: the FeCr15Ni15 alloy after passivation at 0.4 V/SCE for 4500 s (d-f), transpassivation at 1.1 V/SCE (g-i) and the (111) surface after passivation at 0.4 V/SCE for 4500 s (j-l). Evidently, after transpassivation, the oxide film is still rich in Cr in the inner-layer, which exhibits higher atomic fraction of Cr and lower Fe fraction in contrast to that of the passivated counterparts. The values of atomic fraction, at the position corresponding to the maximum of Cr and meanwhile the minimum of Fe (marked by arrows), are used to calculate the Cr/Fe ratio. For each condition, we extracted 21 groups of line-distribution curves corresponding to the variant positions and correspondingly obtained 21 points of Cr/Fe ratio. (m) The cumulative probability analysis on the Cr/Fe ratio confirming the enhanced enrichment of Cr induced by the transpassivation treatment; while the passive films share similar Cr/Fe ratio. In addition to the three types of samples corresponding to the treatment adopted in the present work, the experimental data versus the variety of passivation durations as well as passive potentials are supplemented, further confirming the reliability of the EDS line-distribution analysis.

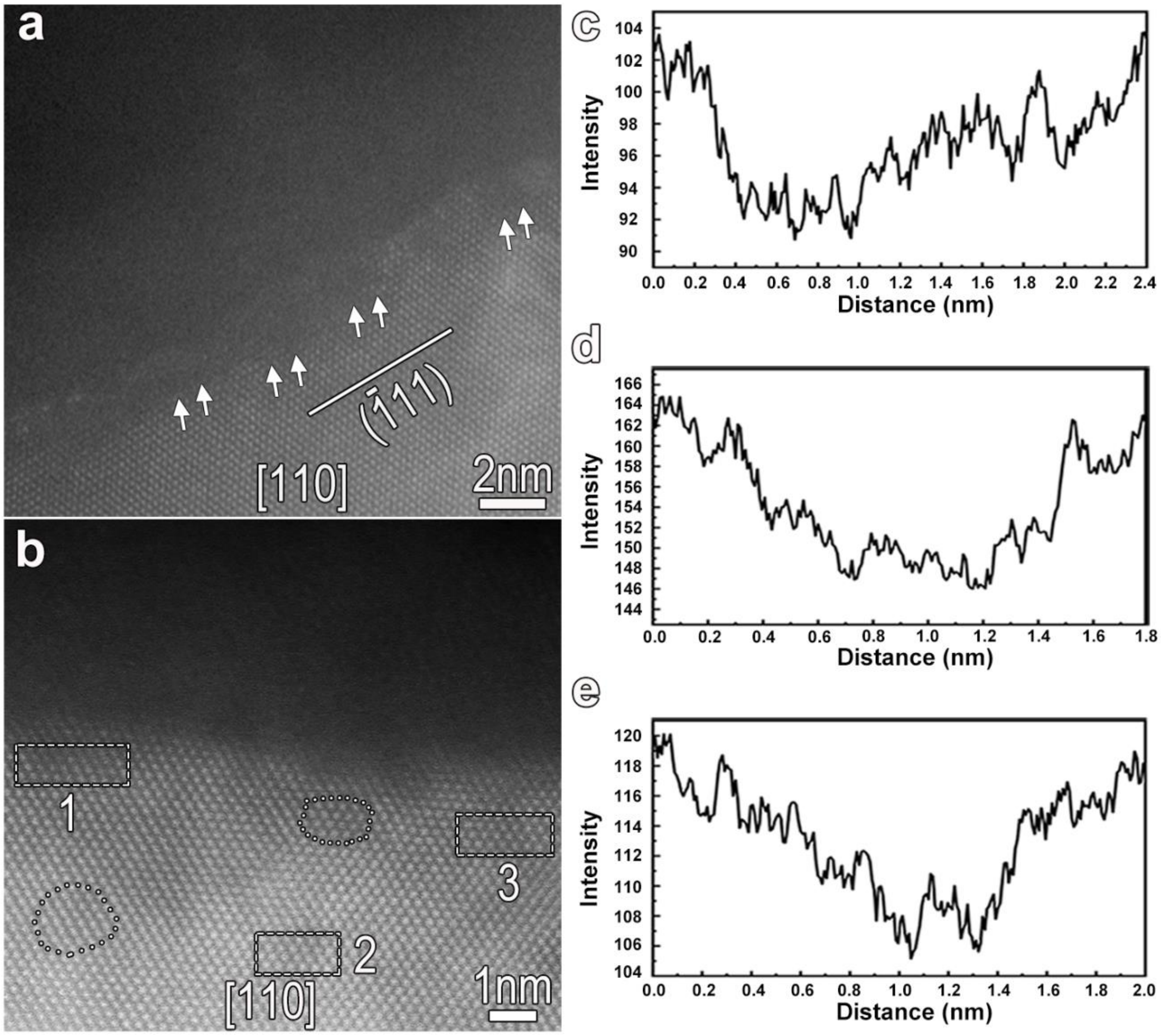

Supplementary Fig. 6. Enhanced dissolution of the matrix under the transpassive potential, leaving behind a large number of metal vacancies. (a, b) HRHAADF-STEM images along the $[110]_{matrix}$ direction, showing some locations at the metal side, with darker-contrast atom columns (labeled by white arrows in a and dotted circles and rectangles 1, 2, 3 in b. (c-e) Intensity profiles corresponding to positions 1 (c), 2 (d) and 3 (e), showing that the lowest intensity corresponds to the darkest atom columns.

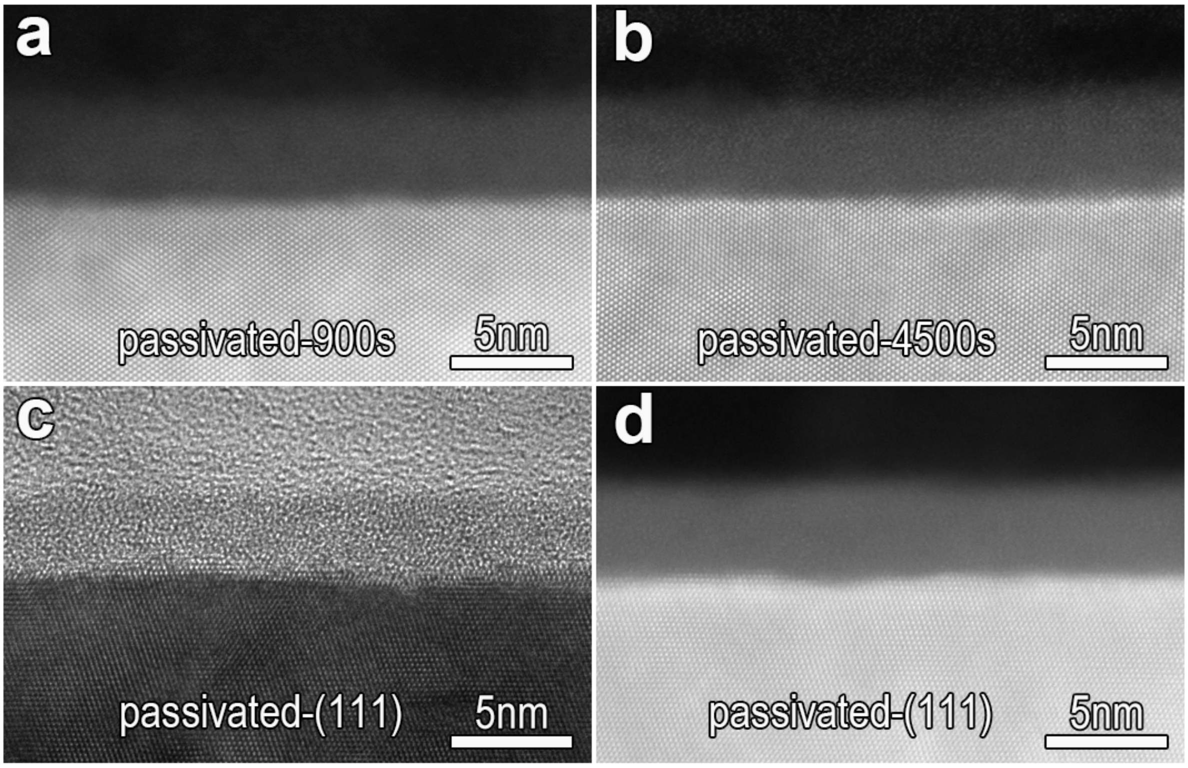

Supplementary Fig. 7. High resolution HAADF-STEM/TEM images showing the passive films on the (111) and (110) share similar microstructure (mostly amorphous). (a-b) High resolution HAADF-STEM images showing the passive films on (110) surface are mostly amorphous. (c-d) High resolution TEM (c) and HAADF-STEM (d) images showing the passive films on the (111) is also mostly amorphous and isn’t crystallized like that of the transpassive film.

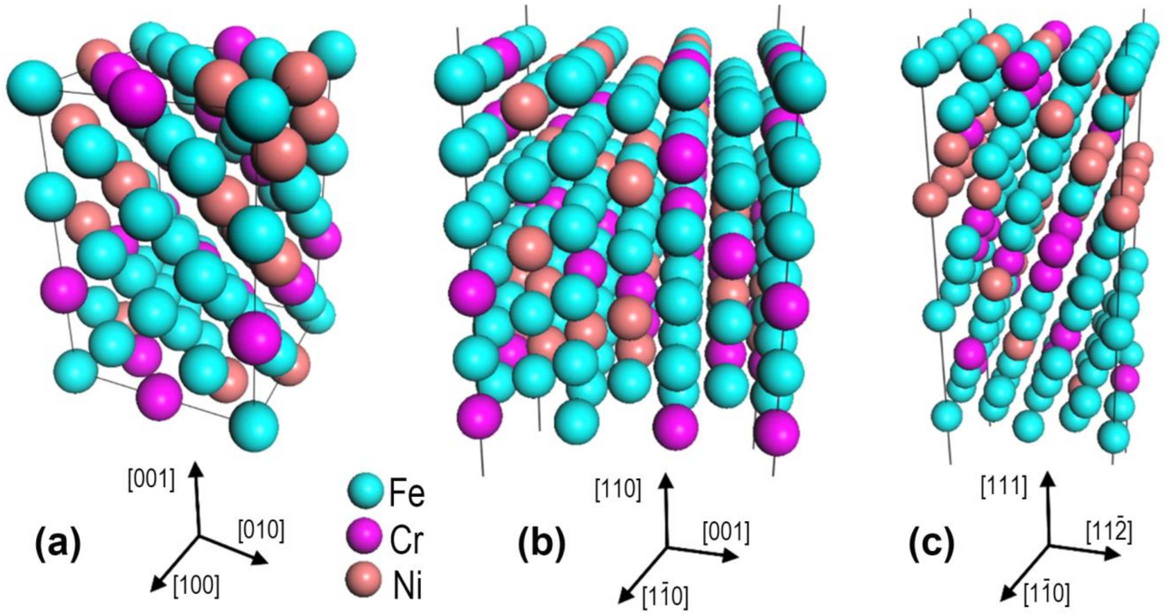

(d)

Surface		1	2	3	4	5	ρ (1/A²)
(110)	E_{surf}(J/m²)	2.981	2.891	2.870	3.011	2.982	0.585
	E_{surf}/ρ (eV)	0.319	0.309	0.307	0.322	0.319	
(111)	E_{surf}(J/m²)	2.629	2.562	2.586	2.685	2.640	0.764
	E_{surf}/ρ (eV)	0.215	0.210	0.212	0.220	0.216	
	I(110)/I(111)	28~75 (average value of 50)					

Supplementary Fig. 8. Simulation of dissolution rate ratios for different crystallographic planes by first-principles calculations. (a-c) Typical atomic structures of the Fe-Cr-Ni alloy showing the bulk model (a) and the slab models of the (110) (b) and (111) (c) surfaces. (d) Data table showing the calculated values of the correlated parameters. The five groups of E_{surf} values were calculated to correspond to the five possible replacements of Fe atoms by Cr and Ni. The current density ratio of the two surfaces ranged from 28 ~ 75 and the averaged ratio is about 50.

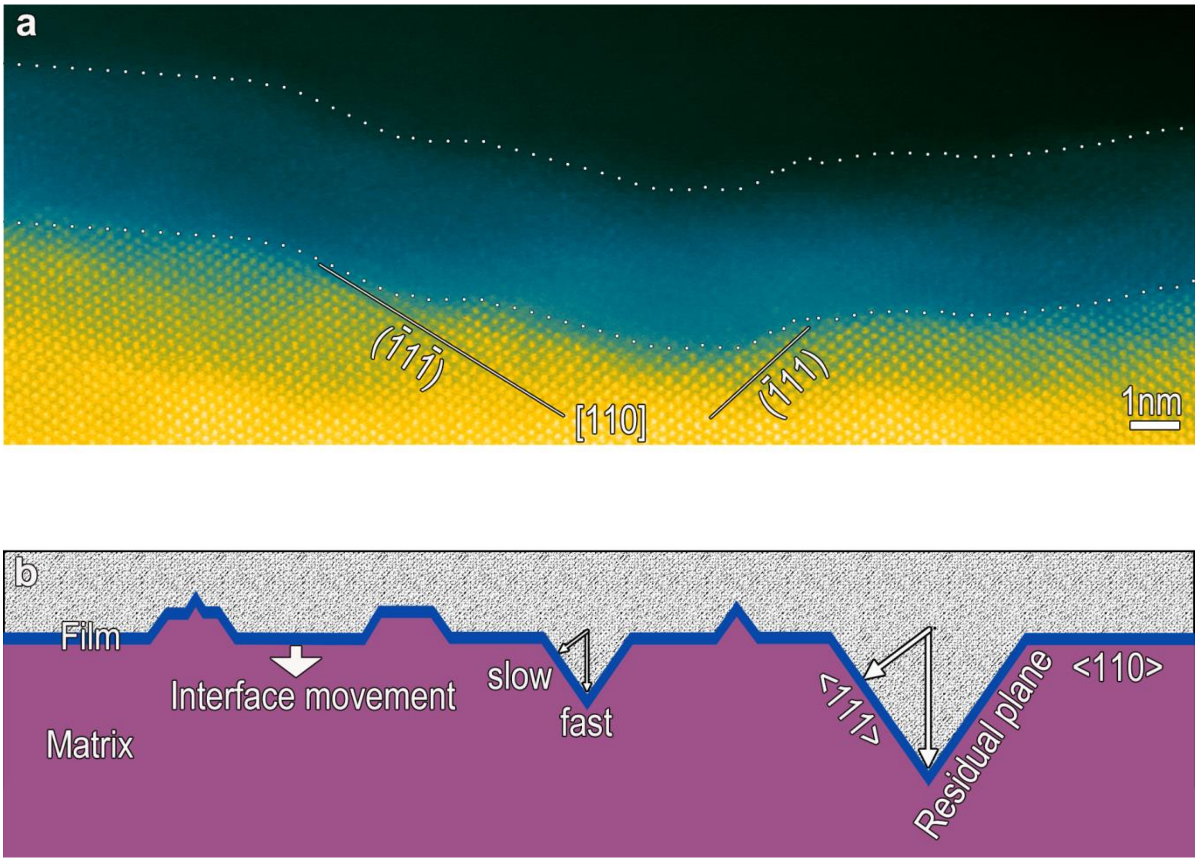

Supplementary Fig. 9. Schematic illustrating the formation of an undulating interface with transpassivation. (a) High resolution HAADF-STEM image showing the transpassivation-induced undulation of the interface with exposure of the close packed plane. (b) Schematic map illustrating the formation of the undulating interface with distinct crystallographic planes, ascribed to the varied dissolution rates along different crystallographic directions. Under transpassive potentials, the dissolution of the FeCr15Ni15 matrix along the <110> direction is much faster than that along the <111> direction, yielding the {111} residual plane at the interface.

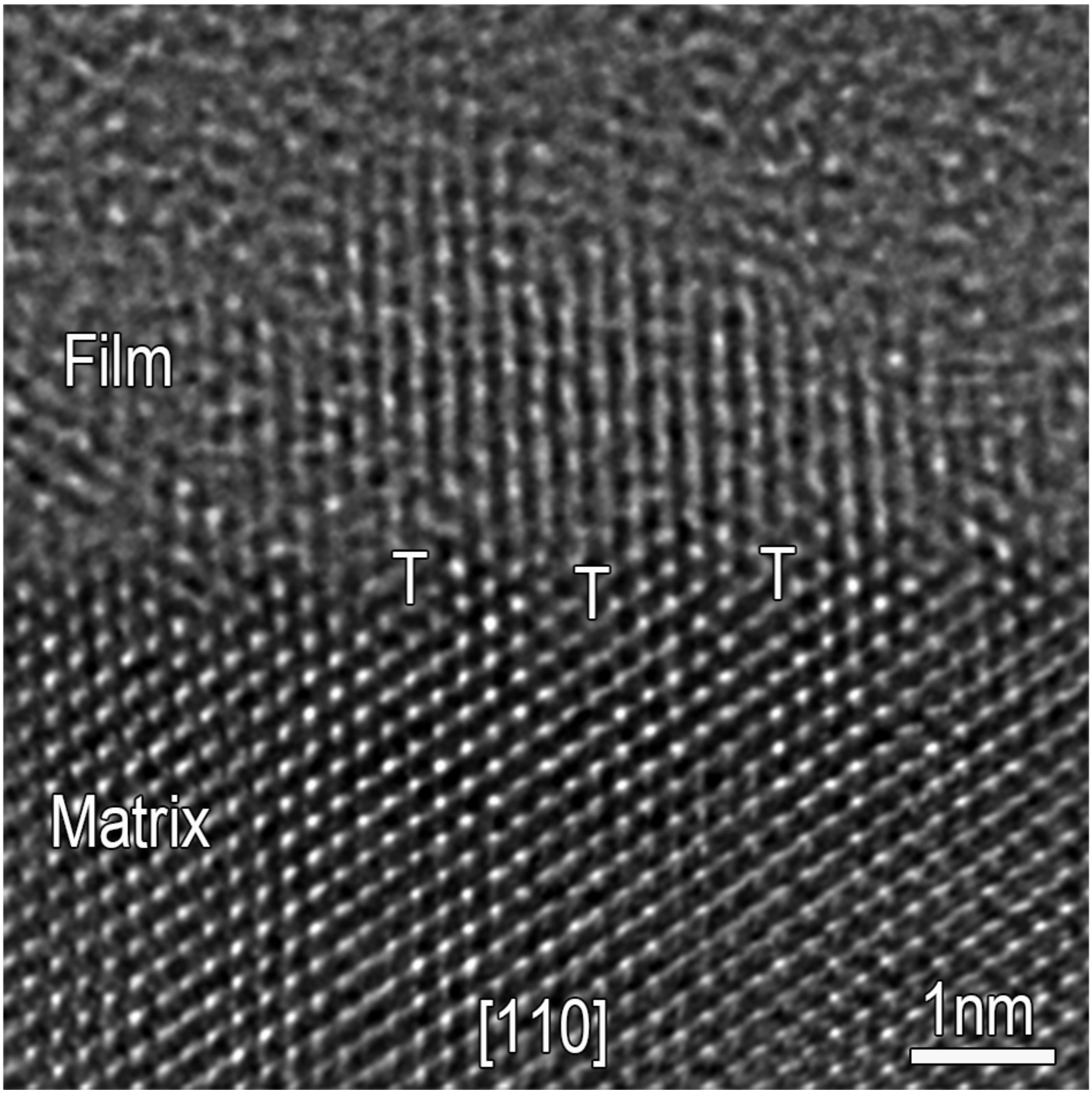

Supplementary Fig. 10. HRTEM image taken along the $[110]_{matrix}$ direction, showing some misfit dislocations at the Me/F interface of the passivated FeCr15Ni15.

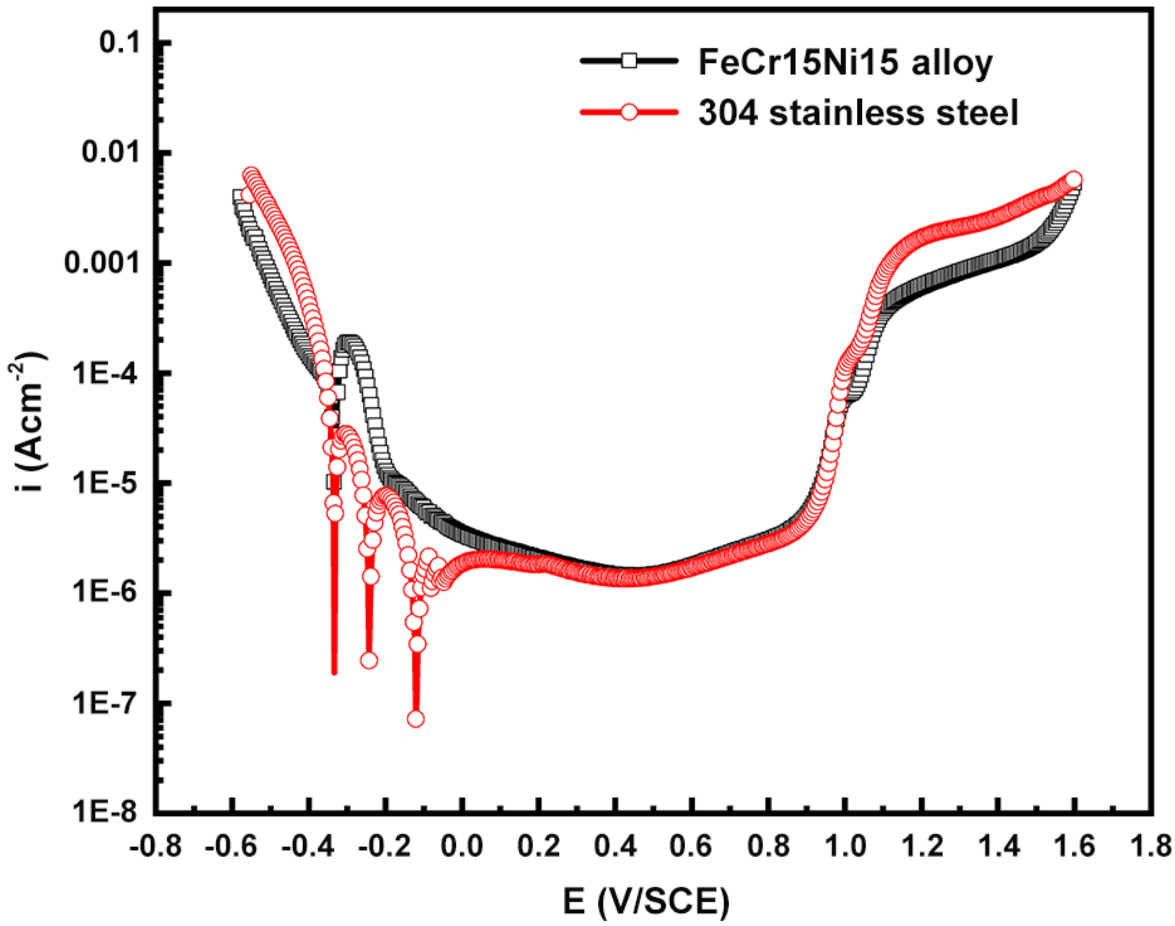

Supplementary Fig. 11. Potentiodynamic polarization curves of the commercial 304 SS and FeCr15Ni15 single crystal alloy in 0.5 mol L^{-1} H_2SO_4 electrolyte.

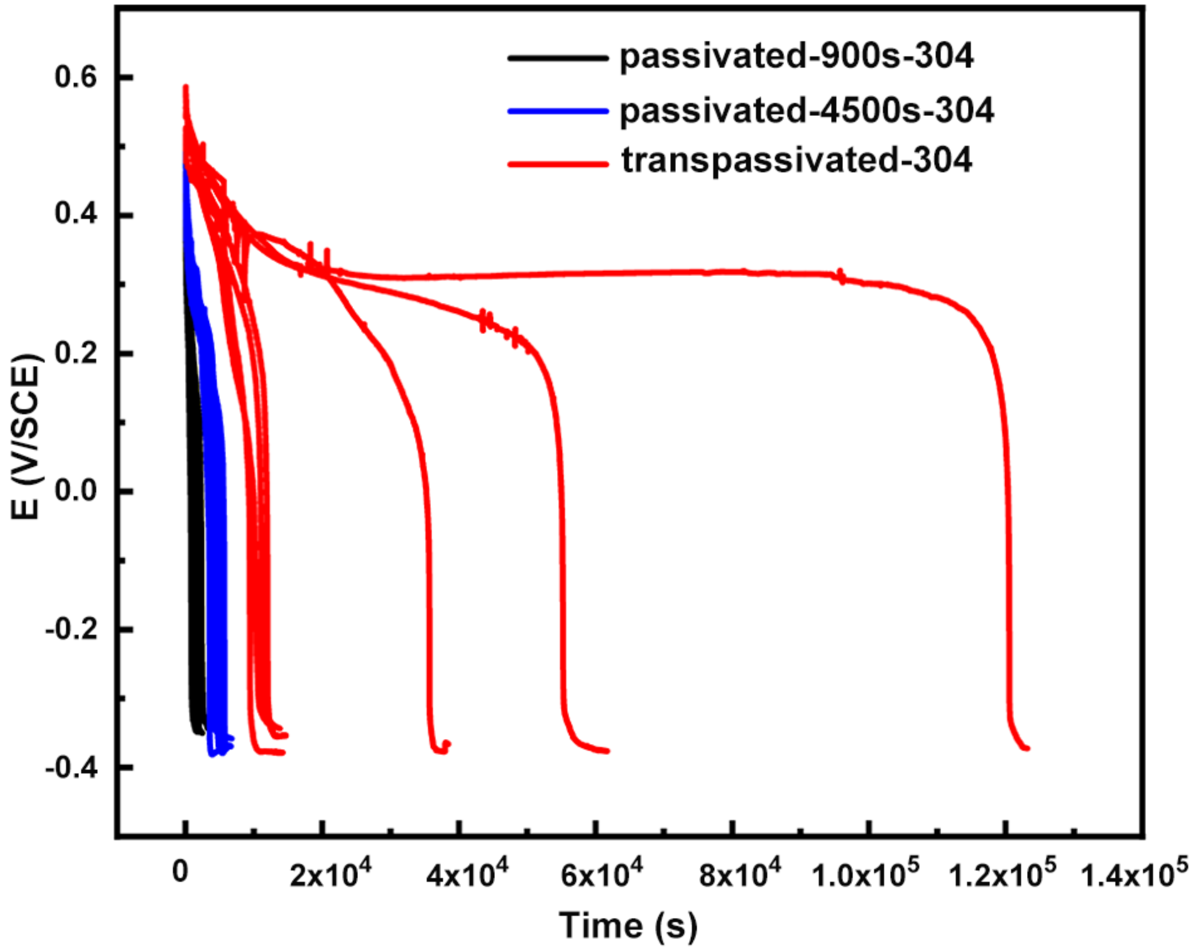

Supplementary Fig. 12. Typical potential decay curves of the three types of 304SS samples (2mm×2mm) in 5.6 mol L^{-1} H_2SO_4 electrolyte at room temperature showing that the transpassivation treatment extends the activation time. The three types of 304SS samples correspond to the same procedure with that of the FeCr15Ni15 single crystal alloy: (1) passivated in 0.5 mol L^{-1} H_2SO_4 electrolyte at 0.4 V/SCE for 900 s (passivated-900s-304); (2) passivated in 0.5 mol L^{-1} H_2SO_4 electrolyte at 0.4V/SCE for 4500 s (passivated-4500s-304); (3) passivated in 0.5 mol L^{-1} H_2SO_4 electrolyte at 0.4 V/SCE for 900 s firstly, and then transpassivated at 1.1 V/SCE for 3600 s (transpassivated-304).

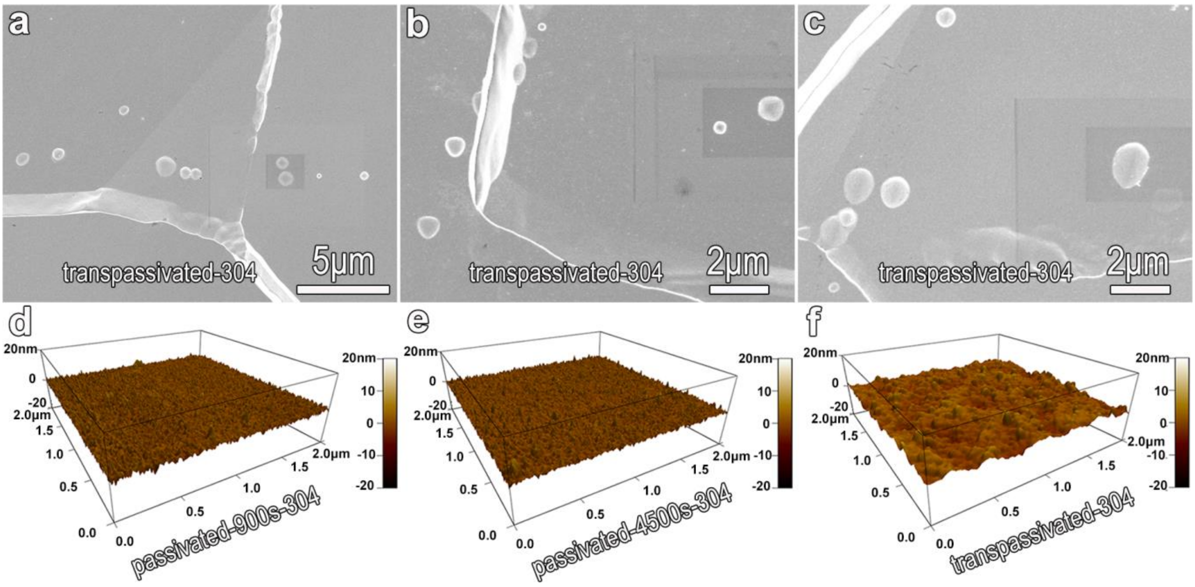

Supplementary Fig. 13. Transpassivation-induced preferential dissolution of the metal matrix at grain boundaries (GBs) and re-configures the metal surface at the interior of grains. (a-c) Zoom-in SEM images of the transpassivated 304 SS surface (2mm×2mm) showing presence of GBs and dispersal of small pits in the interior of the grains. The GBs have gully-like morphology, and the small pits have regular appearance. It is noteworthy that the pits in the single grains have similar appearance, which implies that, although the orientations differ between the grains, the transpassive dissolution of the metal matrix underneath the film proceeds along specific crystallographic orientations leaving the low-energy planes. (d-f) AFM images of the passivated (d, e) and transpassivated (f) surfaces, illustrating that transpassivation treatment roughens the surface, forming a large number of shallow cones, similar to the case of the FeCr15Ni15 single crystal alloy. It is worthwhile to note that the picture f was imaged within a single grain excluding the pits.

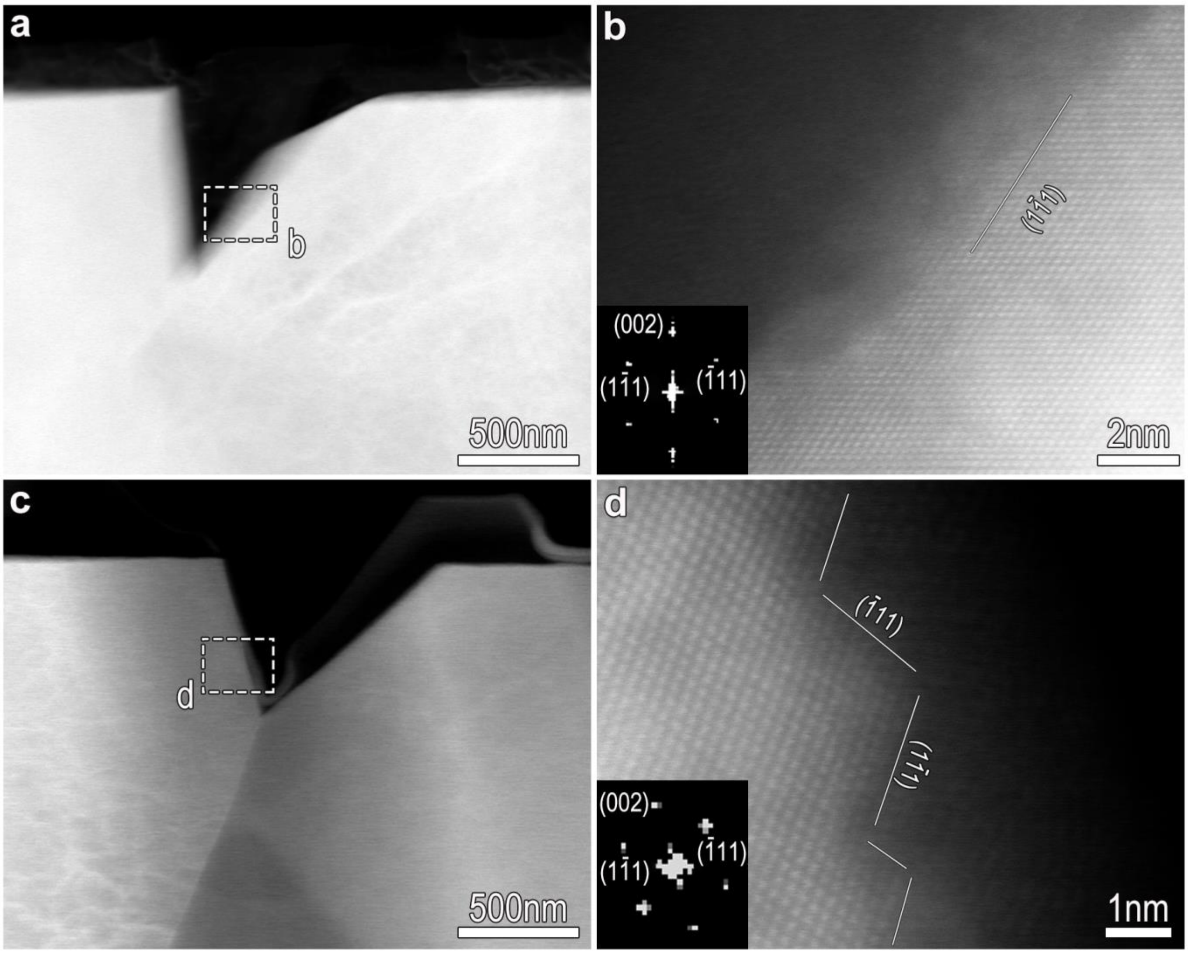

Supplementary Fig. 14. Transpassivation engineers the Me/F interface by forming steep concaves preferentially at GBs. (a) HAADF-STEM image showing a typical steep concave with amplitude in the order of a few hundred of nanometers, formed along the GB after transpassivation treatment. (b) Zoom-in image shows the high resolution HAADF-STEM image of the rectangular labelled region b, located at the wall of the concave in a. The wall is along the close packed {111} plane. (c) HAADF-STEM image showing the deeper concave formed along the GB. (d) Zoom-in image shows the high resolution HAADF-STEM image of the rectangular labelled region d located at the wall of the concave in c. The wall of the concave is composed of highly dense {111} zigzag facets and in such a case the terminal plane might macroscopically deviate somewhat from the {111}. Insets show the Fast Fourier Transform (FFT) images of the high resolution HAADF-STEM image.

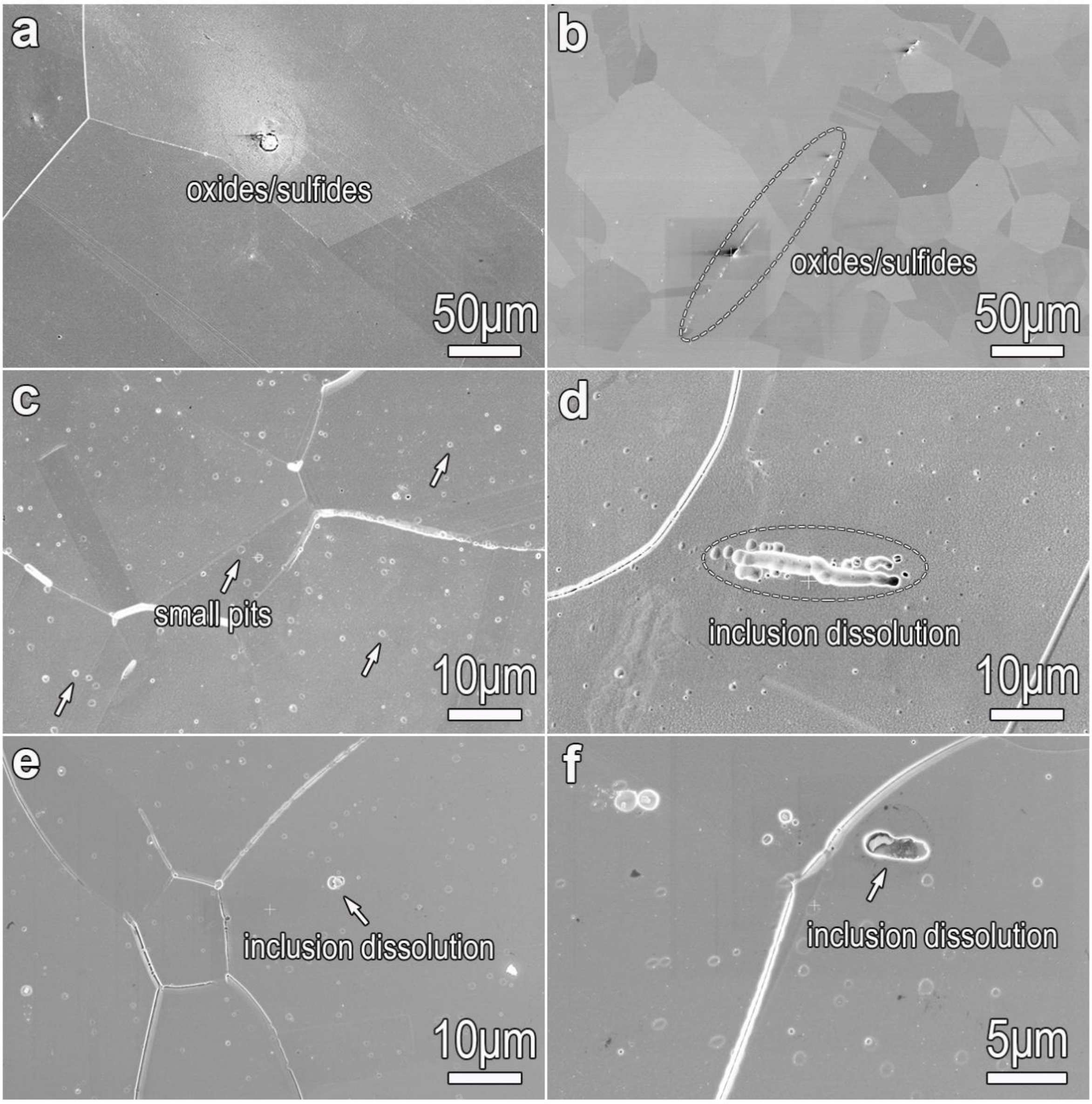

Supplementary Fig. 15. SEM images showing that the coarse inclusions weaken the effect of interface engineering by anodic transpassivation potential. (a) SEM image of the transpassivated 304 SS surface (10mm×10mm) indicates that the evident local corrosion damage occurs at the coarse sulfide-oxide composite inclusions, while the representative morphology of small pits almost does not form in the interior of the grains. (b) SEM image of the electrochemically polished 304 SS surface with small size of inclusions (Nippon steel corporation in Japan). (c-f) Zoom-in SEM images of the transpassivated 304 SS surface (10mm×10mm, Nippon steel corporation in Japan). Although the local dissolution also occurs at small inclusions, the transpassivation rebuilds the GBs into the gully-shaped steep concaves and produces small pits dispersing in the interior of the grains.

Supplementary Note 1

Preparation and orientation determination of the FeCr15Ni15 single crystal. FeCr15Ni15 (wt. %) single crystal alloy was used as matrix on which the passive and transpassive films were formed for the study. The preparation, characterization and orientation determination have been described in our previous work[1]. Here, a brief summary is given. The single crystal alloy was grown by the thermal gradient directional solidification method and has an austenite single phase. By means of single-crystal X-ray diffractometery, two low-index crystallographic orientations [001] and [110] were obtained. Then, the rod was cut into1.3 mm × 2.2 mm ×1.5 mm cuboids, with the two adjacent orthogonal surfaces being (001) and (110) crystallographic planes respectively. Accordingly, three orthogonal surfaces (001), (110) and (-110) were obtained. By means of tilting the clamper device (fixing the single crystal rod) in three dimensions, [111] orientation was also fixed, and thus single crystal sample with an exposure of (111) plane was made.

Supplementary Note 2

Transpassivation treatment. The ground cuboid specimens were polished electrochemically in $HClO_4$ (10 vol. %) + ethyl alcohol (90 vol. %) at 6~10 V voltage, to remove the deformed layer and obtain the smooth surface. Subsequently, the specimens with (110) plane as exposure surface were sealed with thread sealing tape and olefin resin to obtain the electrodes for subsequent transpassivation treatments.

An AUTOLAB PGSTAT302N electrochemical workstation and a traditional three-electrode system were used in electrochemical experiments. The working electrode was the FeCr15Ni15 alloy, Pt was used as counter electrode and a saturated calomel electrode (SCE) as reference electrode. Potentiodynamic polarization measurements were performed firstly (shown in Supplementary Fig. 1), with a scan rate of 20 mV/min, in aerated 0.5 mol L^{-1} H_2SO_4. Thereafter, a suitable passive potential (0.4 V/SCE) was selected to form a passive film, while a transpassive potential (1.1 V/SCE) was used for the transpassivation treatment. Both films were formed potentiostatically. The specimens were depolarized at -1.2 V/SCE for 30 s before potentiostatic polarization, in order to eliminate native oxide formation in air. The potential was then stepped from the corrosion potential to 0.4 V/SCE and maintained for 900 s at this potential, and then further stepped to 1.1 V/SCE and maintained for 1h, during which the current density versus time (i-t) curves were logged.

In order to elucidate the evolution of the nature of passive films induced by transpassivation, distinct films were formed under three different conditions: (1) passivation in 0.5 mol L^{-1} H_2SO_4 electrolyte at 0.4 V/SCE for 900 s (passivated-900s), (2) passivation in 0.5 mol L^{-1} H_2SO_4 electrolyte at 0.4 V/SCE for 4500 s (passivated-4500s), and (3) passivation in 0.5 mol L^{-1} H_2SO_4 electrolyte at 0.4 V/SCE for 900 s initially, and subsequent transpassivation at 1.1 V/SCE for 3600 s (transpassivated). To further elucidate the

significant role of the low-energy {111} planes at the Me/F interface in the corrosion resistance, the FeCr15Ni15 single crystal with the surface of {111} exposure was passivated in 0.5 mol L^{-1} H_2SO_4 electrolyte at 0.4 V/SCE for 4500 s (passivated (111)).

Supplementary Note 3

TEM specimen preparation. The strategy adopted in preparing for TEM visualization of the ultra-thin surface film has been described in detail in our previous work[1]. Briefly, cross-sectional TEM specimens were prepared by bonding two passivated or transpassivated surfaces face-to-face and then thinning by grinding and ion-milling. In the sealing, passivating/transpassivating as well as sealing-tape removal, the surfaces were strictly free of touch and any mechanical damage.

Aberration-corrected transmission electron microscopy, Titan G^2 60-300 microscope and Titan Cubed Themis G^2 300 (fitted with a high-brightness field-emission gun, double Cs corrector, and a monochromator operating at 300 kV; configured with the fast-speed Super-X EDS detector), were used to obtain the high-resolution TEM and HAADF-STEM images as well as Super-X EDS analysis to the surface film.

Supplementary Note 4

Performance evaluation of the FeCr15Ni15 alloy with transpassivation. Potential decay tests were performed to evaluate the resistance of passive and transpassive films to reductive dissolution in acid media. Passive films on the (111) surfaces were also evaluated to further approve the role of the low-energy interface in the corrosion resistance of the film. The four types of samples (described in Supplementary Note 2) were immersed in 5.6 mol L^{-1} H_2SO_4 electrolyte at room temperature, during which the open circuit potential decay with time was recorded, as shown in Fig. 7a. The time corresponding to the abrupt potential decay happening next to the potential plateau, defined as activation time (τ), is usually used to evaluate the resistance of passive films to reductive dissolution[2,3]. Although the time interval corresponding to the abrupt potential drop differs for the different samples, the final potential values attained are almost similar at about -0.35 V, which implies complete removal of the surface film in localized areas and local exposure of the metal matrix.

Five parallel specimens were tested for each treatment condition, and five activation time values (τ) were obtained for each condition. Although the τ values are somewhat scattered, the cumulative probability analysis of τ (Fig. 7b) reveals that transpassivation treatment induced remarkable expansion of the activation time by up to two orders of magnitude beyond that anticipated from non transpassivated counterparts. This further confirms that transpassivation treatment greatly enhanced the resistance to reductive dissolution of the FeCr15Ni15 single crystal alloy.

Similarly, the cumulative probability analysis of pitting potential (E_{pit}) was used to

evaluate the enhancement of pitting resistance after transpassivation treatment. The E_{pit} values were obtained from the potentiodynamic polarization tests in 3.5% NaCl electrolyte at 50 °C. It is found that transpassivation treatment shifts the E_{pit} (the E_{pit} value at $P_{cum} = 0.5$) to noble values by about 120 mV on the average (Fig. 7c-d).

Cumulative probability shown in the vertical axis was calculated by a mean rank method: $P_{cum} = i/(N+1)$, where P_{cum} is the cumulative probability of measured activation time values (τ) or pitting potential (E_{pit}) , i is the order in the total number N (i = 1, 2, 3, …, N).

Supplementary Note 5

Simulation of the dissolution rate ratio for different planes. Electrochemical dissolution rate can be related to the current density (*I*). According to the work of Ma et al[4], the current density *I* of a surface undergoing anodic dissolution at a constant potential is related to the surface energy E_{surf} and surface atom density ρ:

$$I \propto \exp\left(\frac{E_{surf}}{\rho k_B T}\right) \tag{1}$$

where k_B is the Boltzmann constant and T is the absolute temperature (T is taken value of 298.15 K here). The effect of surface vacancies and adsorption is neglected. Thus, the ratio of the current densities for the (110) and (111) surfaces $I_{(110)} / I_{(111)}$ can be written as:

$$\frac{I_{(110)}}{I_{(111)}} = \frac{\exp\left(\frac{E_{surf}(110)}{\rho_{(110)} k_B T}\right)}{\exp\left(\frac{E_{surf}(111)}{\rho_{(111)} k_B T}\right)} = \exp\left[\frac{1}{k_B T}\left(\frac{E_{surf}(110)}{\rho_{(110)}} - \frac{E_{surf}(111)}{\rho_{(111)}}\right)\right] \tag{2}$$

First-principles calculations were performed to calculate values of E_{surf} and ρ of the (110) and (111) surfaces. The surface energy is defined as:

$$E_{surf} = \frac{E_{slab} - N \cdot E_{bulk}}{2S} \tag{3}$$

where E_{slab} is the energy of the slab model and E_{bulk} is the energy per atom of the bulk model; N is e the atom numbers and S is the area of the slab model. The surface atomic density is defined as:

$$\rho = \frac{N_S}{S} \tag{4}$$

where N_S is the number of surface atoms contributing to the surface energy.

Firstly, pure Fe with fcc structure was considered. A series of calculations were carried out for slab models of pure Fe with different thicknesses to determine N_S. For the (110) and (111) surfaces of pure Fe, the top 5 and 4 layers of atoms can be regarded as the surface atoms. Next, we considered the Fe-Cr-Ni alloy. We assume N_S of the two surfaces of the Fe-Cr-Ni alloy are the same as those of pure Fe. The composition of the studied alloy is Fe-Cr15 at%-Ni15 at%. For the sake of simplicity, an approximate composition of Fe-Cr16.7 at%-Ni16.7 at% was adopted in the calculation. The bulk model of the alloy is a 2 × 2 × 3 supercell of a Fe-fcc unit cell and the 1/3 of Fe atoms were selected randomly to be replaced by Cr and Ni atoms. We have built five such models and done full ionic and lattice relaxation to obtain the averaged energy per atom of the Fe-Cr-Ni bulk models. The slab models of the (110) and (111) surfaces of the alloy were constructed by cleaving the Fe-fcc unit cell and substituting Cr and Ni atoms randomly. The lateral sizes of the two slab models are $3a \times 2\sqrt{2}a$ for the (110) surface and $\sqrt{6}a \times \frac{3}{2}\sqrt{2}a$ for the (111) surface, where a is the lattice constant of the Fe-Cr-Ni alloy. Again, five slab models were built for the two surfaces, respectively. Typical atomic structures of the bulk and slab models are shown in Supplementary Fig. 8. After ionic relaxation, the total energies of the slab models were calculated and the corresponding surface energies also obtained (Supplementary Fig. 8). Using Eq. (2), the current density ratio of the two surfaces was calculated. The average ratio was about 50.

Supplementary Note 6

Investigation of transpassivation strategy on commercial metals. In order to examine the validity of this approach to commercially relevant metals, we evaluated the performance of the type 304 austenitic stainless steel (304 SS) with transpassivation treatment compared to the available passivation strategies. A commercial AISI 304 SS, made by Taiyuan Iron and Steel Group Co., Ltd. (TISCO) in China, with a composition of Fe-18.29Cr-8.10Ni-0.061C-0.44Si-1.30Mn -0.006S-0.078P (wt.%), was used. Potentiodynamic polarization curve of the 304 SS shows the almost same passive potential range with the FeCr15Ni15 single crystal alloy (Supplementary Fig. 11). Accordingly, the

same potentials of passivation (0.4V/SCE) and transpassivation (1.1V/SCE) with those of the FeCr15Ni15 single crystal alloy were selected.

In order to examine the role of GBs and inclusions in engineering low-energy interface on metal, two kinds of samples are designated. One features smaller area with 2 mm×2 mm and the other larger with 10 mm×10 mm. For each kind of samples, three types of treatments were conducted: (1) passivation in 0.5 mol L^{-1} H_2SO_4 electrolyte at 0.4 V/SCE for 900 s (passivated-900s-304); (2) passivation in 0.5 mol L^{-1} H_2SO_4 electrolyte at 0.4 V/SCE for 4500 s (passivated-4500s-304) and (3) passivation in 0.5 mol L^{-1} H_2SO_4 electrolyte at 0.4 V/SCE for 900 s initially, and subsequent transpassivation at 1.1 V/SCE for 3600 s (transpassivated-304). The three types of samples with small exposure areas were subjected to potential decay tests in 5.6 mol L^{-1} H_2SO_4 electrolyte at room temperature, during which the open circuit potential decay with time was recorded, as shown in Supplementary Fig. 12. Seven parallel specimens were tested for each treatment condition, and seven activation time values (τ) were obtained for each condition. Although the τ values are somewhat scattered, the cumulative probability analysis of τ (Fig. 9a) reveals that transpassivation treatment induced expansion of the activation time by up to two orders of magnitude beyond that of the commonly available passivation strategies. Meanwhile, we performed the potentiodynamic polarization curve tests in 0.5 mol L^{-1} H_2SO_4 + 3.5% NaCl at 50 °C, and the cumulative probability analysis of pitting potential (E_{pit}) (Fig. 9b-c).

The similar tests were performed on the 304 SS with large exposure areas, and the morphologies after transpassivation treatment were examined (Supplementary Fig. 15).

Supplementary References

[1] Zhang, B. et al. Unmasking chloride attack on the passive film of metals. Nat. Commun. 9, 2559 (2018).

[2] Kim, Y. *et al.* Electrochemical analysis on the potential decay behavior of Fe-20Cr stainless steels in sulfuric acid solution. *Electrochim. Acta* **266**, 1-6 (2018).

[3] Uhlig, H. H. & King, P. F. The flade potential of iron passivated by various inorganic corrosion inhibitors. *J. Electrochem. Soc.* **106**, 1-7 (1959).

[4] Ma, H. et al. First-principles modeling of anisotropic anodic dissolution of metals and alloys in corrosive environments. Acta Mater. 130, 137-146 (2017).
