# Supplementary material for: Enhanced corrosion resistance by engineering crystallography on metals
Source: Nat Commun. 2022 Feb 7;13:726. doi: 10.1038/s41467-022-28368-8 (PMC8821614; doi:10.1038/s41467-022-28368-8)
Supplement: Supplementary file 2 — Peer Review File [file 41467_2022_28368_MOESM2_ESM.pdf]

Editorial Note: This manuscript has been previously reviewed at another journal that is not operating a transparent peer review scheme. The manuscript was considered suitable for publication without further review at *Nature Communications*.
